# Mass Spectrometry-Based Network Analysis Reveals New Insights Into the Chemodiversity of 28 Species in *Aspergillus* section *Flavi*

**DOI:** 10.3389/ffunb.2021.719420

**Published:** 2021-08-11

**Authors:** Xinhui Wang, Karolina Subko, Sara Kildgaard, Jens C. Frisvad, Thomas O. Larsen

**Affiliations:** ^1^Department of Biotechnology and Biomedicine, Technical University of Denmark, Lyngby, Denmark; ^2^Food Machinery and Chemical (FMC) Agricultural Solutions, Hørsholm, Denmark; ^3^Section for Ecology and Evolution, Department of Biology, University of Copenhagen, Copenhagen, Denmark

**Keywords:** *Aspergillus* section *Flavi*, cyclopiazonic acid, tenuazonic acid, fumifungin, chemical diversity, LC-MS/MS, molecular networking

## Abstract

*Aspergillus* section *Flavi* includes some of the most famous mycotoxin producing filamentous fungi known to mankind. In recent years a number of new species have been included in section *Flavi*, however these species have been much less studied from a chemical point of view. In this study, we explored one representative strain of a total of 28 fungal species in section *Flavi* by systematically evaluating the relationship between taxonomy and secondary metabolites with LC-MS/MS analysis for the first time and dereplication through an in-house database and the Global Natural Product Social Molecular Networking (GNPS) platform. This approach allowed rapid identification of two new cyclopiazonic acid producers (*A. alliaceus* and *A. arachidicola*) and two new tenuazonic acid producers (*A. arachidicola* and *A. leporis*). Moreover, for the first time we report species from section *Flavi* to produce fumifungin and sphingofungins B-D. Altogether, this study emphasizes that the chemical diversity of species in genus *Aspergillus* section *Flavi* is larger than previously recognized, and especially that understudied species are prolific producers of important mycotoxins such as fumi- and sphingofungins not previously reported from this section. Furthermore, our work demonstrates Global Natural Product Social (GNPS) Molecular Networking as a powerful tool for large-scale chemotaxonomic analysis of closely related species in filamentous fungi.

## Introduction

*Aspergillus* section *Flavi* comprises a large number of species, many of which have a significant impact on human health and the society (Varga et al., 2011). *Aspergillus flavus* and *Aspergillus parasiticus* are two of the most studied species, because they are predominant species responsible for contamination of crops and food, and because of their capacity to produce aflatoxins (Hedayati et al., 2007; Rank et al., 2012; Frisvad et al., 2019; Uka et al., 2019), which are famous carcinogenic, teratogenic and mutagenic mycotoxins (Bräse et al., 2009; Norlia et al., 2019). Among them, AFB_1_ is the most potent one classified as a group 1 human carcinogen by the International Agency for Research on Cancer (IARC) (Claeys et al., 2020). Therefore, it is crucial to identify aflatoxin and other mycotoxin producers, in order to design management strategies for avoiding their contamination of crops and monitor threats to human health.

On the other hand non-toxinogenic species such as *A. oryzae* and *A. sojae* are used for production of sake, soya sauce and other fermented foods. Furthermore, *A. oryzae* is an industrial workhorse for the production of enzymes (Ferrão et al., 2017; Frisvad et al., 2018). Due to their importance, Kjærbølling and co-workers recently sequenced 19 genomes spanning section *Flavi* and compared 23 species, which, from a genetic point of view, demonstrate great diversity in their secondary metabolism (Kjærbølling et al., 2020). To group the increasing number of new species in section *Flavi* according to their phylogenetic relationships, Houbraken and co-workers have recently introduced new series in the classification of genus *Aspergillus*, grouping the current 36 species in section *Flavi* into the eight series *Alliacei, Avenacei, Bertholletiarum, Coremiiformes, Flavi, Kitamyces, Leporum* and *Nomiarum* (Gilchrist et al., 2020; Houbraken et al., 2020). However, only a few studies have systematically categorized metabolites from the now many species in section *Flavi* from a chemical perspective in order to explore potential mycotoxins or valuable compounds (Uka et al., 2020). Therefore, we here propose a mass spectrometric (MS) based workflow to comprehensively analyze the metabolites among the species in section *Flavi* and further systematically evaluate their chemotaxonomic relationships. We hypothesize that several understudied species in this section are also likely to be the producers of known mycotoxins, and thereby pose a potential risk to contaminate food and agricultural products and to cause health problems in humans (Ferrão et al., 2017; Frisvad et al., 2018). More specifically, in this report we have applied a GNPS molecular networking based strategy consisting of taxonomic information and an in-house fungal metabolite library search with the aim of identifying new metabolite families and finding new mycotoxin producers from a set of 28 strains representing individual species in *Aspergillus* section *Flavi* (Kildgaard et al., 2014). Molecular networking was developed as a promising approach for the analysis of MS fragmentation data and for visualization of a spectral similarity map between measured metabolites (Yang et al., 2013; Aron et al., 2020). It is part of a central component of the Global Natural Products Social (GNPS) molecular networking platform for performing dereplication against a large, community-acquired reference library of spectra (Wang et al., 2016). The underlying assumption in a molecular networking analysis is that structurally related molecules will likely share comparable MS/MS fragmentation patterns. Therefore, similar compounds will gather in the final networks to create clusters of analogs (Watrous et al., 2012). Molecular networking is a powerful tool for the organization and visualization of large datasets. Furthermore, it offers the possibility of multiple annotations including different kinds of data such as spectrometric, taxonomic, or biological features (Crüsemann et al., 2017; Nothias et al., 2018; Olivon et al., 2019). Despite the above-mentioned advantages, it must be pointed out that the success of a molecular networking-based dereplication relies greatly on the quality and the availability of MS/MS data (Fox Ramos et al., 2019). Moreover, the dereplication process by molecular networking itself annotates only a limited number of nodes because of the limited amount of MS files for fungal secondary metabolites in the GNPS library. To this end, an in-house MS/MS library was implemented in this report to efficiently target important mycotoxins as well as unidentified analogs across species in section *Flavi* (Kildgaard et al., 2014). With this combined approach, all the producers of each investigated metabolite were shown by the molecular networks and important mycotoxins that have not been found previously in this section were detected, uncovering a more comprehensive description of the chemical profiles of species in this important section. Altogether, this study highlights novel species-specific and series-specific metabolite production relationships and enables the prioritization of strains for future MS-guided natural product discovery projects.

## Materials and Methods

### General Experimental Procedures

All solvents were purchased from Sigma-Aldrich (Steinheim, Germany). The formic acid used was from Fluka (for LC-MS). Water was purified using a Milli-Q system (Millipore, Bedford, MA, USA).

### Culturing and Extraction

The spore suspension (1 × 10^6^ ~ 10^7^ spores/mL) was used to inoculate the CYA growth media, which contained the following ingredients (g/L Milli-Q water): yeast extract (5 g), Czapek dox broth (35 g), agar (20 g), ZnSO_4_.7H_2_O (0.01 g), CuSO_4_.5H_2_O (0.005 g). Briefly, the section *Flavi* strains (Supplementary Table S1) were grown as three point inoculations for seven days at 25°C on CYA in the dark. Subsequently three plugs (6 mm inner diameter) were taken across the colony. One ml of isopropanol: ethyl acetate (1:3 v/v) with 1% formic acid was added for extraction of secondary metabolites and the mixture was sonicated for 1 h. The liquid sample was transferred to another tube and evaporated. Methanol (500 μl) was then added to dissolve the extracts and the samples were sonicated for 10 min. Samples were then centrifuged for 3 min, and afterwards 150 μl of the supernatant was transferred to HPLC vials for analysis. All samples were prepared and analyzed in an identical manner.

### Data-Dependent LC-ESI-HRMS/MS Analysis

Ultra-high-performance liquid chromatography–diode array detection–quadrupole time-of-flight mass spectrometry (UHPLC–DAD–QTOFMS) was performed on an Agilent Infinity 1290 UHPLC system (Agilent Technologies, Santa Clara, CA, USA) equipped with a diode array detector. Separation was achieved on a 250 × 2.1 mm i.d., 2.7 μm, Poroshell 120 Phenyl Hexyl column (Agilent Technologies, Santa Clara, CA) held at 60°C. The sample, 1 μL, was eluted at a flow rate of 0.35 mL min^−1^ using a linear gradient from 10% acetonitrile (LC-MS grade) in Milli-Q water buffered with 20 mM formic acid increasing to 100% in 15 min, staying there for 2 min before returning to 10% in 0.1 min. Starting conditions were held for 3 min before the following run.

Mass spectrometry (MS) detection was performed on an Agilent 6545 QTOF MS equipped with Agilent Dual Jet Stream electrospray ion source (ESI) with a drying gas temperature of 160°C, a gas flow of 13 L min^−1^, sheath gas temperature of 300°C and flow of 16 L min^−1^. Capillary voltage was set to 4000 V and nozzle voltage to 500 V in positive mode. MS spectra were recorded as centroid data, at an *m/z* of 100–1700, and auto MS/HRMS fragmentation was performed at three collision energies (10, 20, and 40 eV), on the three most intense precursor peaks per cycle. The acquisition rate was 10 spectra s^−1^. Data were handled using Agilent MassHunter Qualitative Analysis software (Agilent Technologies, Santa Clara, CA). Lock mass solution in 70 % MeOH in water was infused in the second sprayer using an extra LC pump at a flow of 15 μL/min using a 1:100 splitter. The solution contained 1 μM tributylamine (Sigma-Aldrich) and 10 μM Hexakis (2, 2, 3, 3-tetrafluoropropoxy) phosphazene (Apollo Scientific Ltd., Cheshire, UK) as lock masses. The [M + H]^+^ ions (*m/z* 186.2216 and 922.0098, respectively) of both compounds were used. In-house fungal metabolite library search was done as described by Kildgaard et al. (2014).

### Molecular Networking and Data Analysis

All MS/MS data were converted from Agilent MassHunter data files (.d) to the mzXML file format with MS-Convert and uploaded to the GNPS Web platform (http://gnps.ucsd.edu.) for molecular networking analysis using Classic mode (Watrous et al., 2012; Wang et al., 2016). Species belonging to the same series were analyzed together as one group in GNPS, resulting in five groups. In addition, eight standard samples were included in the sixth group. Different parameters (cosine, minimum matched peaks) were evaluated to determine the best networking conditions. A cosine score of 1 indicates identical spectra, while a cosine score of 0 indicates no similarity. Finally, the data were filtered by removing all MS/MS fragment ions within +/– 17 Da of the precursor *m/z*. MS/MS spectra were window filtered by choosing only the top 6 fragment ions in the +/– 50 Da window throughout the spectrum. The precursor ion mass tolerance was set to 0.1 Da and an MS/MS fragment ion tolerance of 0.02 Da. A network was then created where edges were filtered to have a cosine score above 0.69 and more than six matched peaks. Further, edges between two nodes were kept in the network if, and only if, each of the nodes appeared in each other's respective top 10 most similar nodes. Finally, the maximum size of a molecular family was set to 100, and the lowest scoring edges were removed from molecular families until the molecular family size was below this threshold. The spectra in the network were then searched against the GNPS spectral libraries. The library spectra were filtered in the same manner as the input data. All matches kept between network spectra and library spectra were required to have a score above 0.69 and at least 6 matched peaks. The generated molecular network was visualized using Cytoscape 3.7.2 (38) (Smoot et al., 2011), whereby consensus spectra are presented as nodes connected by edges to aligning nodes.

All MS/MS spectra of dereplicated compounds from Supplementary Table S2 were also annotated and deposited in the GNPS library, and all data files and workflows can be found at the links below: GNPS molecular networking job: https://gnps.ucsd.edu/ProteoSAFe/status.jsp?task=1479da25db9a49b79bbfe4cfc7084e51. MASSIVE: doi: 10.25345/C5GZ1W.

## Results

### Generation of the Multi-Informational Molecular Networks

In order to comprehensively investigate the chemical diversity of section *Flavi*, 28 strains of each representing individual species from five series in genus *Aspergillus* section *Flavi* were three point inoculated on Czapek Yeast Agar (CYA). Extracts (isopropanol / ethyl acetate, 1:3, v/v) of the cultures were made after seven days, analyzed by HPLC-HRMS/MS using positive electrospray iononization mode. Next, the obtained fragmentation data was organized by molecular networking from GNPS platform for discovery of potentially novel analogs of known metabolites or identification of possible new mycotoxin producers in *Aspergillus* section *Flavi*.

**Figure 1:**
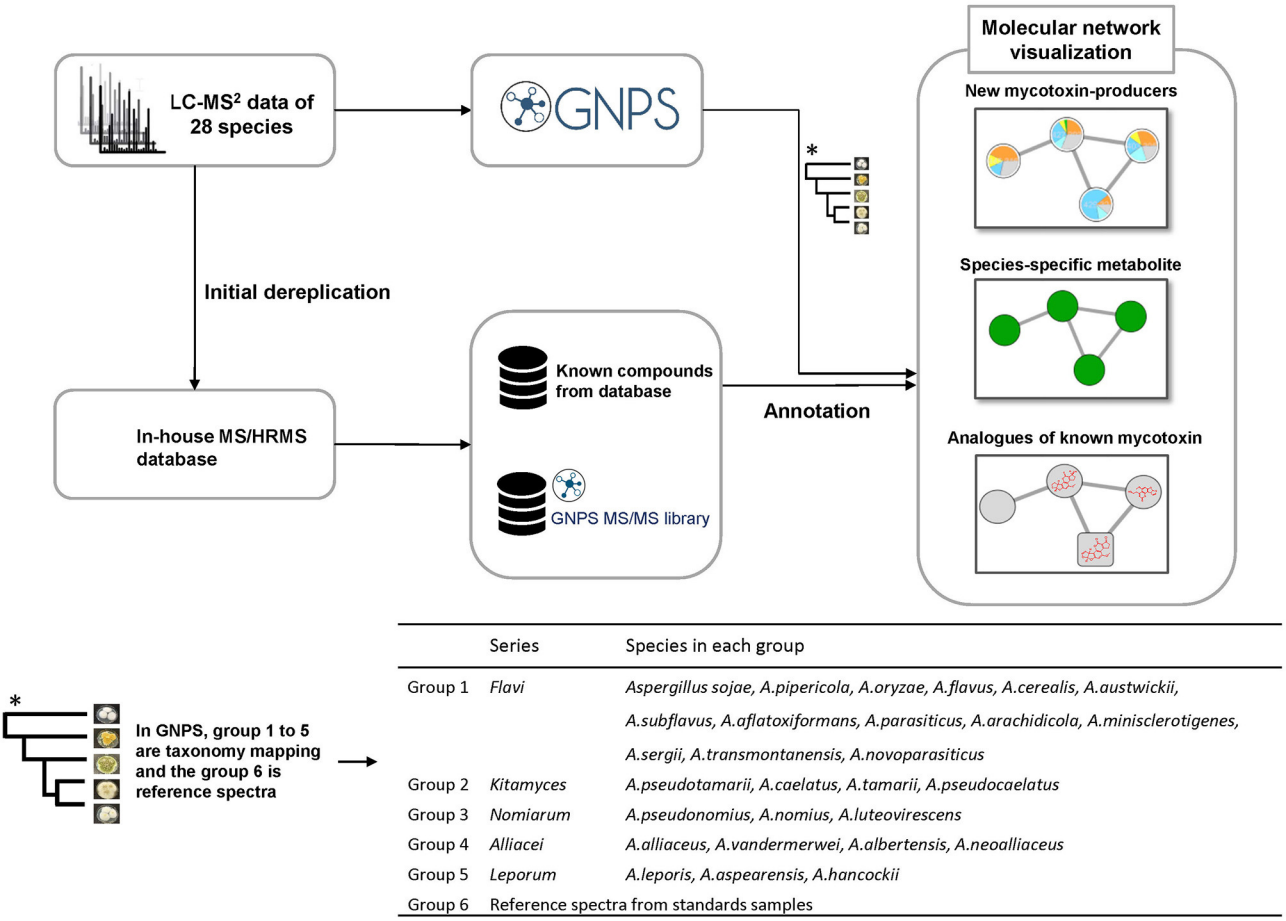
Workflow procedure used in this study.

Molecular networking was mapped with the taxonomic information from the recent report of Houbraken and co-workers (Houbraken et al., 2020), where they introduced a new series classification in *Aspergillus*. In this work we explored the metabolites of the species in series *Alliacei, Flavi, Kitamyces, Leporum* and *Nomiarum* (Figure 1). Each group was assigned a specific color tag. The generated pie chart does not represent quantitative information from the data because it relies only on MS/MS spectral counts. In this way the molecular networking algorithm groups the fragmented ions into clusters using an algorithm to compare the similarity of the fragmentation spectra. When a compound is marked, other analogs with similar structures will also be found because of the MS/MS similarity.

By the help of the initial dereplication step against the GNPS libraries we identified 11 known compounds, including aflatoxin G_1_ and B_1_, ochratoxin A and *aspergillicins*. However, many important metabolites known to be produced by species in section *Flavi* could not be recognized or did not exist in the GNPS library, thus supporting the interest of dereplication by an in-house database (Kildgaard et al., 2014).

For the dereplication of previous known compounds from extracts, the MS/HRMS spectra were searched against the in-house library using Agilent MassHunter PCDL manager (Agilent Technologies) (Kildgaard et al., 2014). Twenty-six compounds (Supplementary Table S2) were found in this step and from these eight important compounds, including aflatoxin B_3_, O-methylsterigmatocystin, ochratoxin B, cyclopiazonic acid, fumifungin, aflatrem, desertorin A, desertorin B (mycotoxins or unique metabolites in this section) were chosen and submitted to GNPS as a starting point, called “seeds,” in order to explore structurally similar molecules in molecular networking. In this way they served as the initial focal points in the network processed from a mixture of unknown compounds using MS/MS spectra (Figure 1).

Altogether, the generated molecular network consisted of 2,155 nodes connected with 2,394 edges with 45.75 % of the nodes organized into a total of 180 molecular clusters comprising two or more nodes each. The self-looped nodes were attributed to unrelated molecules that did not have any structurally related molecules in the samples.

### Analysis of Known Natural Product Molecular Families in the Molecular Network

**Figure 2:**
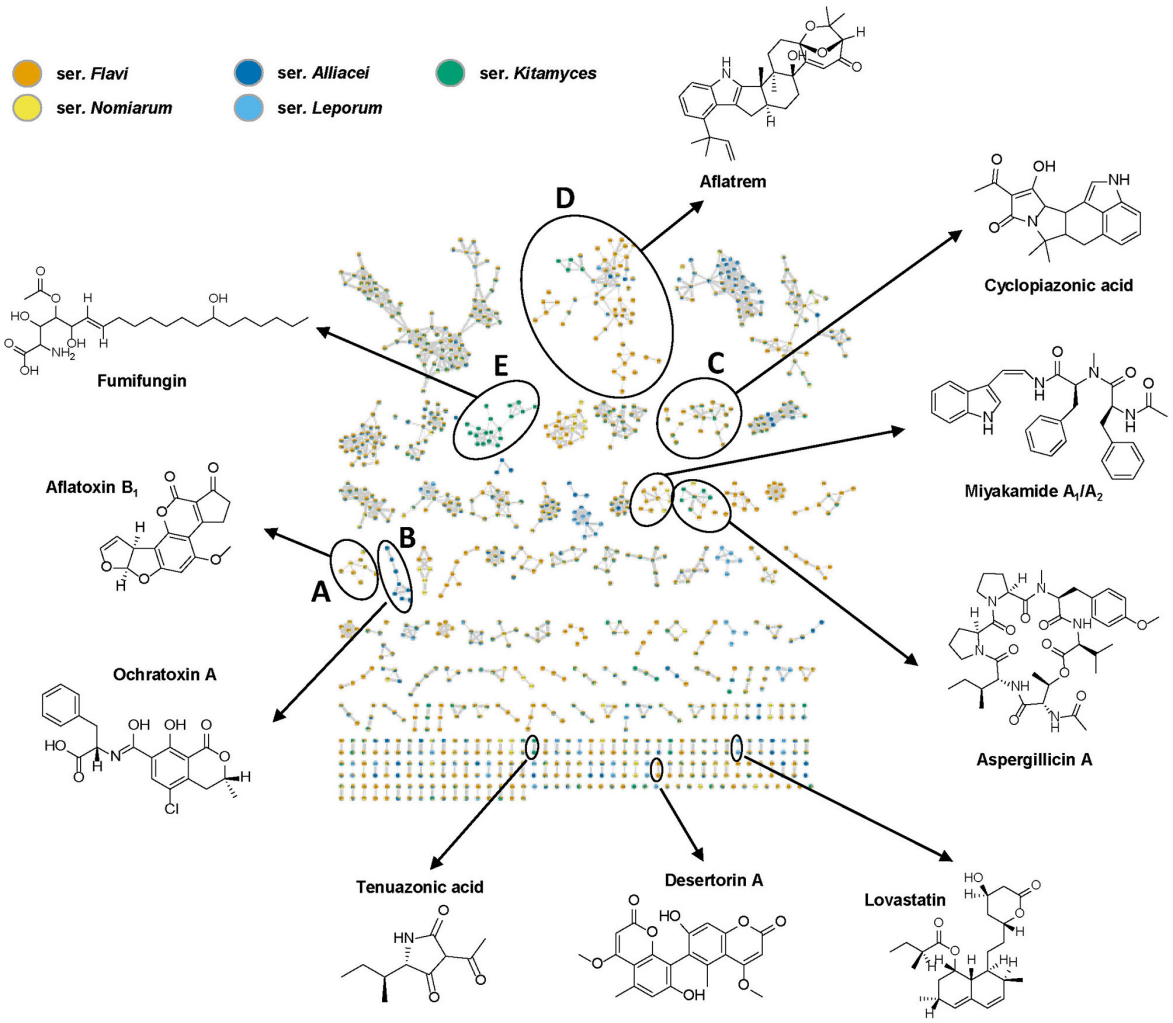
Molecular network of all generated extracts and the standard compounds from the in-house database, including representative structures from 10 known types of compounds. The orange nodes represent ions detected from series *Flavi*; green nodes represent ions detected from series *Kitamyces*; yellow nodes represent ions detected from series *Nomiarum*; blue nodes represent ions detected from series *Alliacei*, while light blue nodes represent ions detected from series *Leporum*. Color-assigned according to their series. Only clusters containing at least two nodes are shown. The detailed view for clusters A**–**E are presented in Figures 3–6.

As expected, molecular networking generated from the 28 species of the section *Flavi* extracts combined with in-house database analysis allowed the dereplication of several known type of compounds such as aflatoxins, ochratoxins, cyclopiazonic acids, aflatrems, tenuazonic acids, lovastatins and miyakamides (Figure 2), among which five clusters (A–E) appeared to be particularly interesting, due to their importance as the mycotoxins and bioactive compounds. The producers of each dereplicated metabolite are outlined in Table 1 and the overall dereplicated compounds are listed in Supplementary Table S2.

**Table 1:** Overview of fungal secondary metabolites detected in this study and their producing species in *Aspergillus* section *Flavi*.

**Molecular Families**	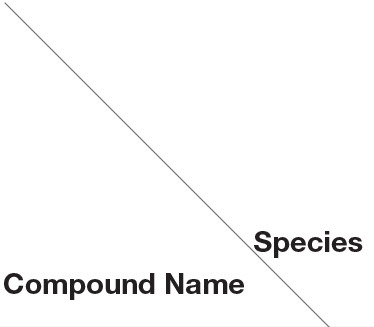	***A.sojae***	***A.pipericola***	***A.oryzae***	***A.flavus***	***A.cerealis***	***A.austwickii***	***A.subflavus***	***A.aflatoxiformans***	***A.parasiticus***	***A.arachidicola***	***A.minisclerotigenes***	***A.sergii***	***A.transmontanensis***	***A.novoparasiticus***	***A.pseudotamarii***	***A.caelatus***	***A.tamarii***	***A.pseudocaelatus***	***A.pseudonomiae***	***A.nomiae***	***A. luteovirescens***	***A.alliaceus***	***A.vandermerwei***	***A.albertensis***	***A.neoalliaceus***	***A.leporis***	***A.aspearensis***	***A.hancockii***	***Abundance***
**Aflatoxins**	**Aflatoxin B1**		+		+	+	+			+	+	+	+	+					+			+								**11**
	**Aflatoxin B3**		+			+	+			+		+	+	+								+								**8**
	**Aflatoxin G1**		+			+	+			+	+	+	+	+					+	+		+								**11**
	**Aflatoxin GM2**		+			+	+																							**3**
	**OMST** ^**a**^		+		+	+	+			+	+	+	+	+					+			+								**11**
	**Sterigmatocystin**					+	+												+	+										**4**
	**Dihydroaflatoxicol**		+			+	+						+	+								+								**6**
**Ochratoxins**	**Ochratoxin A**																						+	+	+	+				**4**
	**Ochratoxin B**																						+		+	+				**3**
	**Ochratoxin beta**																						+		+	+				**3**
	**CTK3E9855**																						+		+					**2**
**CPAs**	**CPA** ^**b**^		+		+	+	+		+		+	+	+			+		+	+				+						+	**13**
	**Speradine A**		+			+	+		+							+		+	+				+							**8**
	**Speradine F**		+			+	+		+			+				+		+	+											**8**
	**2-oxo-CPA** ^**b**^				+	+	+		+									+	+										+	**7**
	**3-OH-speradine A**		+			+	+		+							+		+	+											**7**
**Fumifungins**	**Fumifungin**															+	+	+	+											**4**
	**Sphingofungin B**															+	+	+	+											**4**
	**Sphingofungin C**															+	+	+	+	+										**5**
	**Sphingofungin D**															+	+	+	+											**4**
**Aflatrems**	**Aflatrem**		+			+	+	+				+	+																	**6**
	**Paspalinine**		+			+	+	+				+	+														+	+		**8**
	**Penerpenes**		+			+	+	+				+	+														+	+		**8**
	**Asperindoles**																											+		**1**
	**14-hydroxypaspalinine**																						+		+	+				**3**
	**Paspaline**							+					+														+	+		**4**
**Aflavinines**	**Aflavinine**		+					+				+	+													+		+		**6**
	**14-hydroxyaflavinine**							+				+	+																	**3**
	**Dihydroxyaflavinine**							+					+																	**2**
**Tenuazonic acids**	**Tenuazonic acid**										+					+	+	+	+	+		+					+			**8**
	**Valine-tenuazonic acid**										+					+	+	+	+			+								**6**
**Aspergillicins**	**Aspergillicin A**										+		+				+		+			+								**5**
	**Aspergillicin B**										+		+				+		+											**4**
	**Aspergillicin C**										+		+				+		+											**4**
	**Aspergillicin E/F**						+			+					+															**3**
	**Aspergillicin G**				+	+	+																							**3**
**Miyakamides**	**Miyakamide A1/A2**	+								+				+	+						+									**5**
	**Miyakamide Valine** ^**c**^										+			+	+					+										**4**
	**Miyakamide B1/B2**	+												+	+						+									**4**
	**Aspergillamide A/B**	+		+					+	+	+			+	+					+	+									**9**
	**Oryzamide A1/A2**			+																										**1**
**Desertorins**	**Desertorin A**		+			+	+						+																	**4**
	**Desertorin B**					+	+						+																	**3**
**Aspergillic acids**	**Aspergillic acid**	+						+													+									**3**
	**Flavacol**																							+						**1**
**Lovastatins**	**Lovastatin acid**																											+		**1**
	**Lovastatin analog**																											+		**1**
	**Lovastatin/mevinolin**																											+		**1**
**Parasiticolides**	**Parasiticolide A**							+		+	+																			**3**
	**Deacetylparasiticolide A**			+																										**1**
**Sphingosins**	**Phytosphingosine**	+	+	+	+	+	+	+	+	+	+	+	+	+	+	+	+	+	+	+	+	+	+	+	+	+	+	+	+	**28**
**TMC**	**TMC-95A**																+		+											**2**
**Ustilaginoidins**	**ustilaginoidin C**	+							+	+	+			+	+	+	+	+	+		+	+								**12**
**Avenaciolides**	**(-)-Canadensolide**														+	+							+	+	+	+				**6**
**Diketopiperazines**	**Ditryptophenaline**				+				+	+					+	+	+		+											**7**
**Ergot alkaloids**	**Ergokonin B**		+	+		+	+	+		+		+	+		+	+	+	+		+		+	+						+	**16**
**Heptelidic acids**	**Heptelidic acid**			+			+	+	+																					**4**
**Kojic acids**	**Kojic acid**	+	+	+	+		+					+								+	+	+	+	+	+		+			**13**
**Chrysogines**	**Chrysogine**	+								+	+			+	+		+			+	+	+								**9**

*The rows indicate the known metabolites found in this study and the columns indicate the name of species. Abundance indicates the number of species producing the compound in this study. a OMST: O-methylsterigmatocystin. b CPA: Cyclopiazonic acid. c Miyakamide Valine: Miyakamide analogs with Valine*.

**Figure 3:**
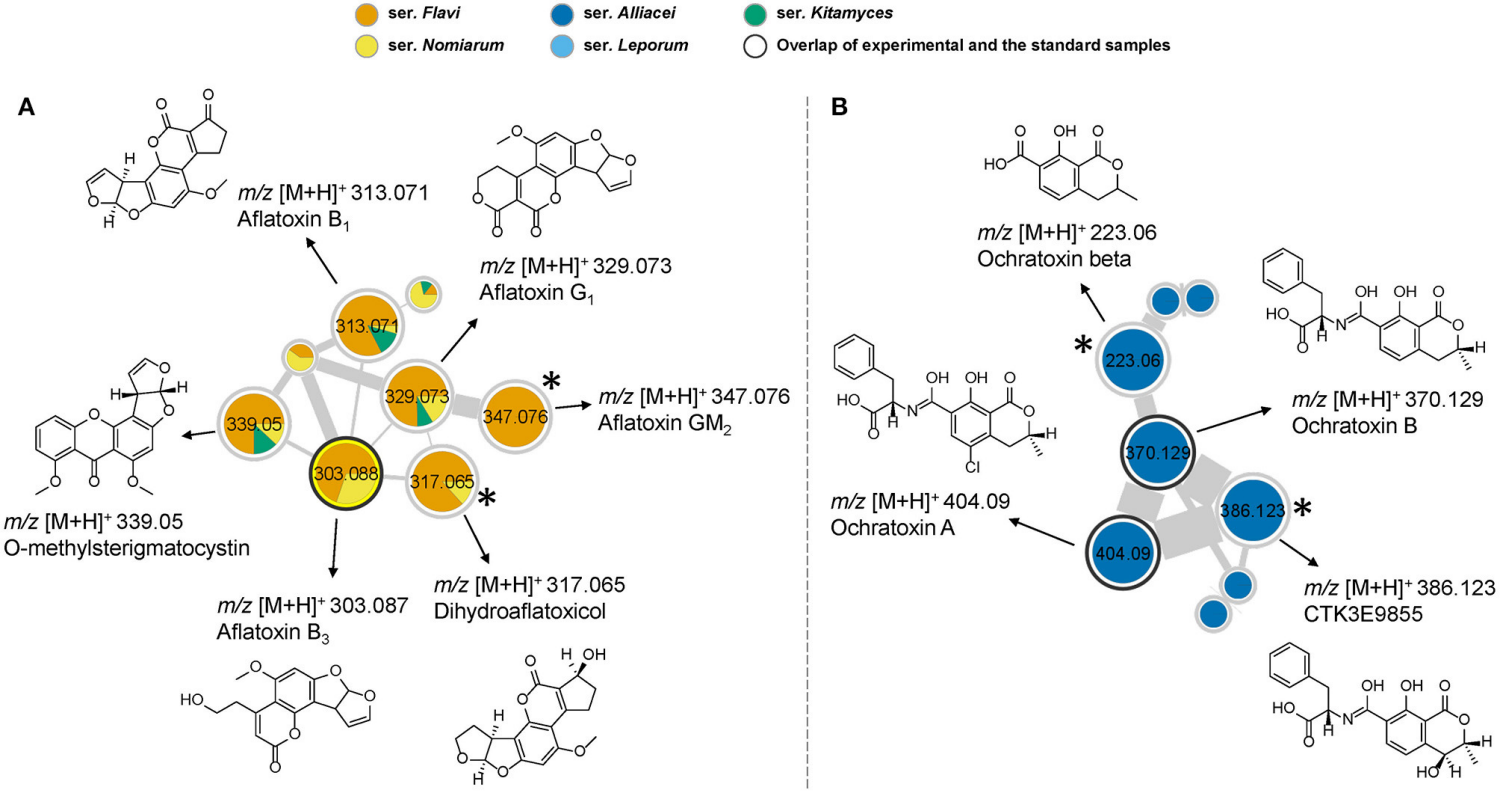
**(A)** Molecular network cluster of aflatoxins. **(B)** Cluster of ochratoxins and their analogs. Nodes with bold outlined black circles are the overlap of molecules and the standard compounds, while the nodes marked with asterisk are the molecules found from the structural relationship shown in molecular networks.

First of all, the network detected the presence of aflatoxin B_1_, B_3_, G_1_, O-methylsterigmatocystin, putative GM_2_ and dihydroaflatoxicol in several samples, as they are shown in the same cluster due to their structural similarity (Figure 3A). Among the 28 studied species in section *Flavi, A. pipericola, A. cerealis, A. austwickii* produced all of the six detected aflatoxins. In addition, *A. parasiticus, A. arachidicola, A. minisclerotigenes, A. sergii, A. transmontanensis, A. pseudocaelatus* and *A. luteovirescens* produced both B- and G type of aflatoxins. Moreover, *A. flavus* produced aflatoxin B_1_ and *A. pseudocaelatus* produced aflatoxin G_1_. All the aflatoxin B_1_ producers had the ability to produce O-methylsterigmatocysin, which is the immediate aflatoxin precursor. These results are all in agreement with previous literature reports (Frisvad et al., 2019).

Ochratoxin A is one of the most abundant food-contaminating mycotoxins and is known to exhibit nephrotoxic, immunosuppressive, teratogenic and carcinogenic properties (Storari et al., 2012). In our study, the nodes of ochratoxin A and B were readily identified by overlapping with the reference spectra. A close look of the network indicated the presence of related analogs in the same cluster, which were putatively assigned as CTK3E9855 and ochratoxin beta based on the comparison of fragmentation patterns (Figure 3B). Interestingly, ochratoxins were only identified from species in the series *Alliacei* that on the other hand did not produce aflatoxins in good agreement with the most recent literature (Houbraken et al., 2020).

**Figure 4:**
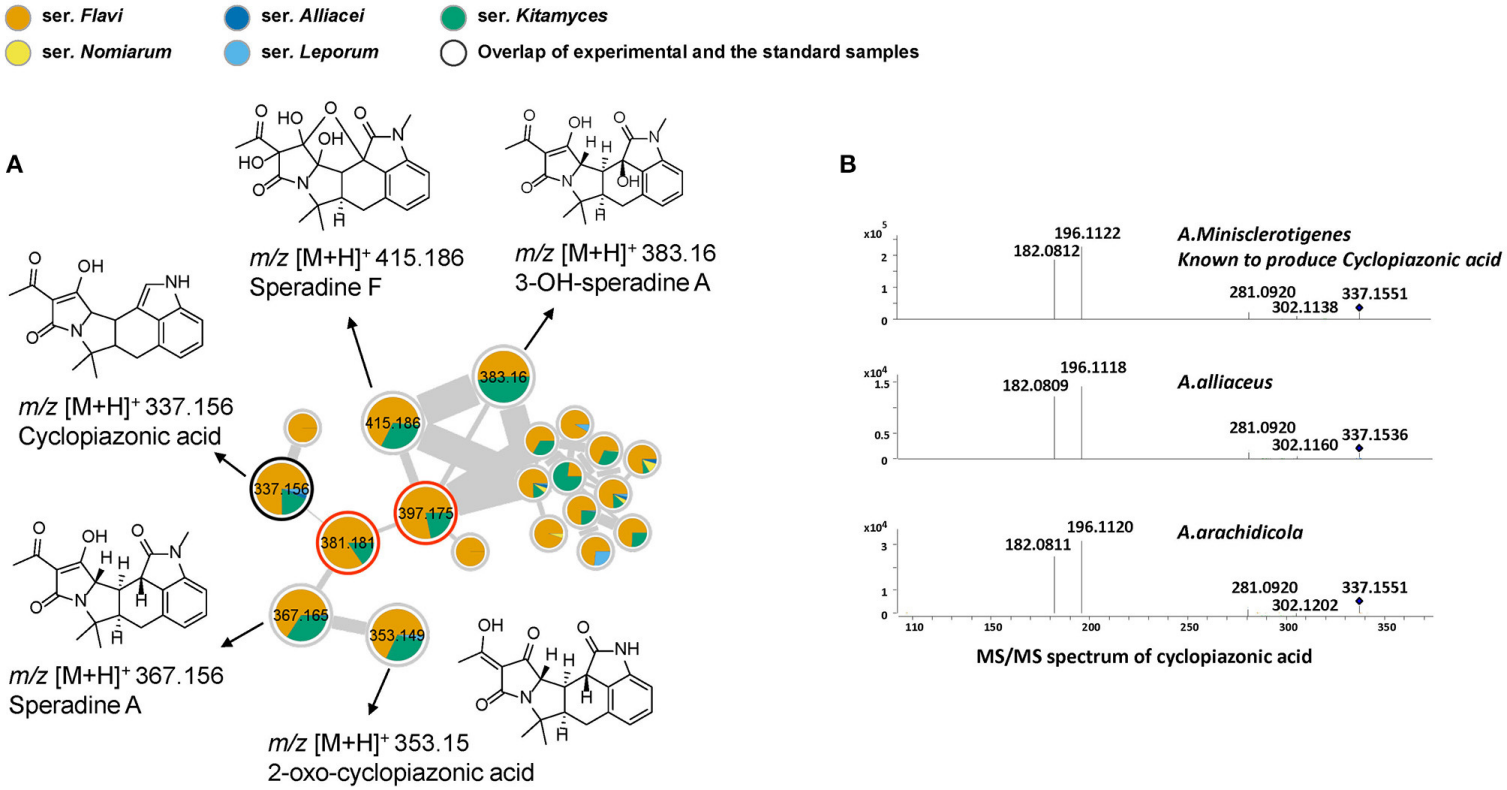
**(A)** Nodes of the compounds related to the cyclopiazonic acid biosynthetic family. Nodes with bold outlined black circles are the overlap of molecules and the standard compounds, the nodes marked with asterisk are the molecules found from the structural relationship shown in molecular networks and the nodes with red outline represent the potentially novel compounds. **(B)** MS/MS spectrum of cyclopiazonic acid (CPA). *A. minisclerotigenes* is the cyclopiazonic acid-producer according to the previous study (Chang et al., 2009). *A. alliaceus* and *A. arachidicola* confirmed as two new CPA producing species by comparing the MS/MS spectra of both strains with known cyclopiazonic acid producer.

Cyclopiazonic acid (CPA) is another important mycotoxin that can be produced by several filamentous fungi used in food fermentations. In addition, CPA is a specific inhibitor of Ca^2+^ stimulated ATPase in the intracellular Ca^2+^ storage sites (Seidler et al., 1989). The main known producers of CPA in section *Flavi* are *A. aflatoxiformans, A. austwickii, A. cerealis, A. flavus, A. minisclerotigenes, A. oryzae, A. pipericola, A. pseudocaelatus, A. pseudotamarii, A. sergii and A. tamarii* (Ferrão et al., 2017). In this study, we notably found that additional species could also produce CPA. In the cluster cyclopiazonic acids (Figure 4A), cyclopiazonic acid (CPA) was first identified by overlapping with the reference spectrum, with structurally similar speradine A characterized by comparison with spectra from our in-house database. A close look of these nodes indicated that 13 strains can produce CPA, among them *A. alliaceus* and *A. arachidicola*, two species that have never previously been reported as CPA producers. This result was further verified from the raw LC-MS data by comparing the spectra of CPA in both strains with the known cyclopiazonic acid producer *A. minisclerotigenes* (Figure 4B). In addition, by comparing with the literature data and exploring the fragment spectra, three other structural related analogs in the same cluster could be identified as 2-oxo-cyclopiazonic acid, speradine F and 3-OH-speradine A (Uka et al., 2017). Furthermore, the exploration of this cluster subsequently allowed the targeting of two potential novel speradines with *m/z* 381.181 [C_22_N_2_O_4_H_24_]^+^ and 397.175 [C_22_N_2_O_5_H_24_]^+^ that did not show any hits when searched against the commercial databases Antibase and Reaxys (Laatsch, 2010)^1^.

**Figure 5:**
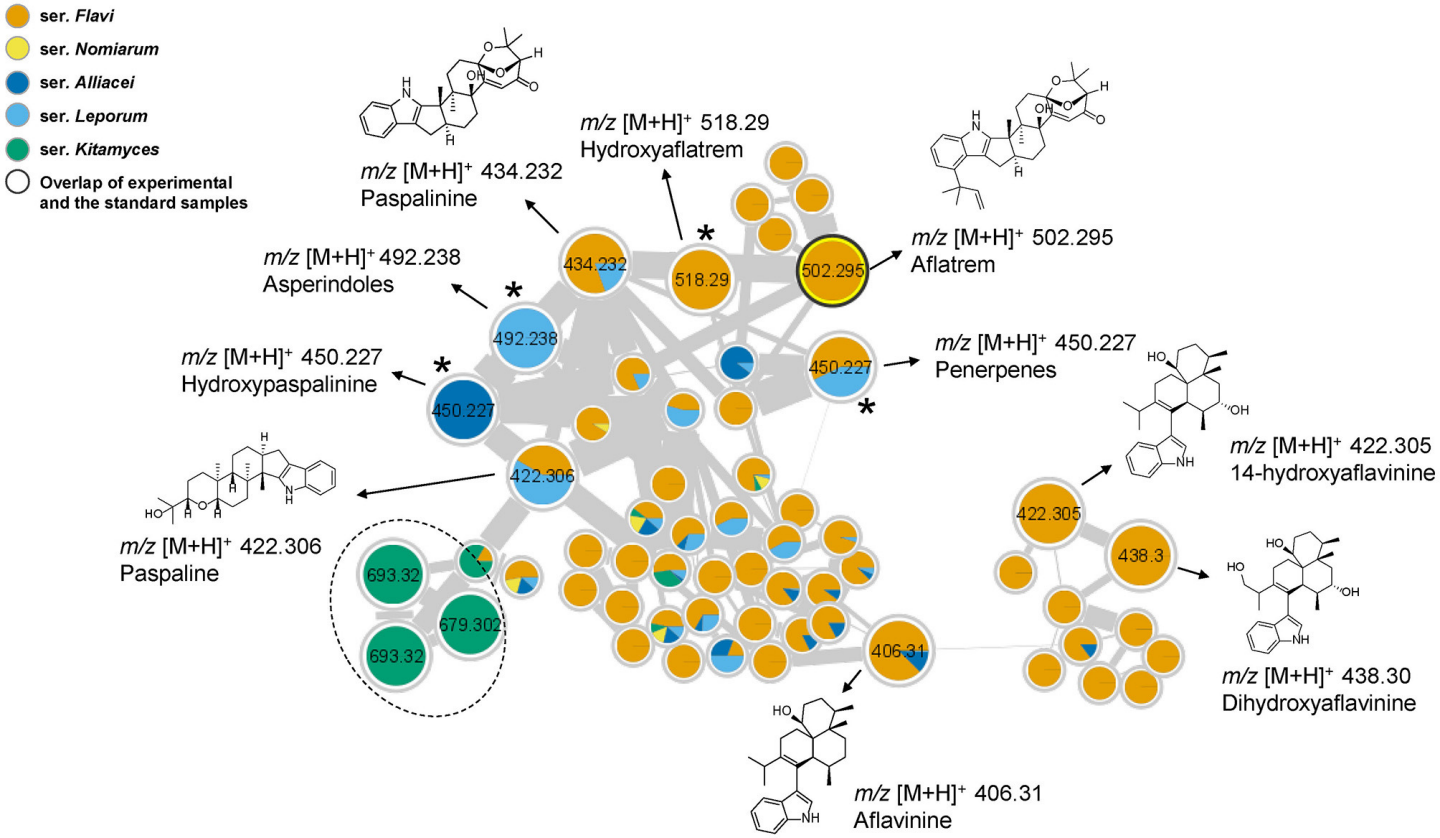
Cluster of aflatrems and related compounds. Nodes with bold outlined black circles are the overlap of molecules and the standard compounds, the nodes marked with asterisk are the molecules found from the structural relationship shown in molecular networks. The products inside the dotted line are only produced in series *Kitamyces*.

Aflatrem is an indole-diterpene produced by *A. flavus* with very potent tremorgenic toxicity (Nicholson et al., 2009), while another indole-diterpene, aflavinine, has been identified as an anti-insectant (Raj Joshi and Adhikari, 2020). Those two compounds and their analogs where observed in the same cluster because of their structural similarity (Figure 5). Among them, the node of aflatrem was firstly identified by overlapping with a reference spectrum. In addition, the molecular network revealed the presence of aflatrem biosynthetic intermediates, including paspaline and paspalinine by comparing the data with in-house database. A close look of the related nodes also identified aflavinine, 14-hydroxyaflavinine and 14, 25-dihydroxyaflavinine by comparing the data with the literature (Rank et al., 2012). The rest of the structural related analogs could be tentatively assigned as asperindoles, penerpenes, hydroxypaspalinine, and hydroxyaflatrem by comparing the fragmentation patterns with identified compounds in the same cluster. However, without the authentic standards we could not confirm the precise identity of those compounds. Interestingly, we found the precursor mass of *m/z* 693.32 in this cluster is unique in series *Kitamyces* and might serve as a new chemical marker to distinguish this series from others in section *Flavi*.

### Discovery of Undescribed Classes of Molecules in Section *Flavi* by Molecular Networking

**Figure 6:**
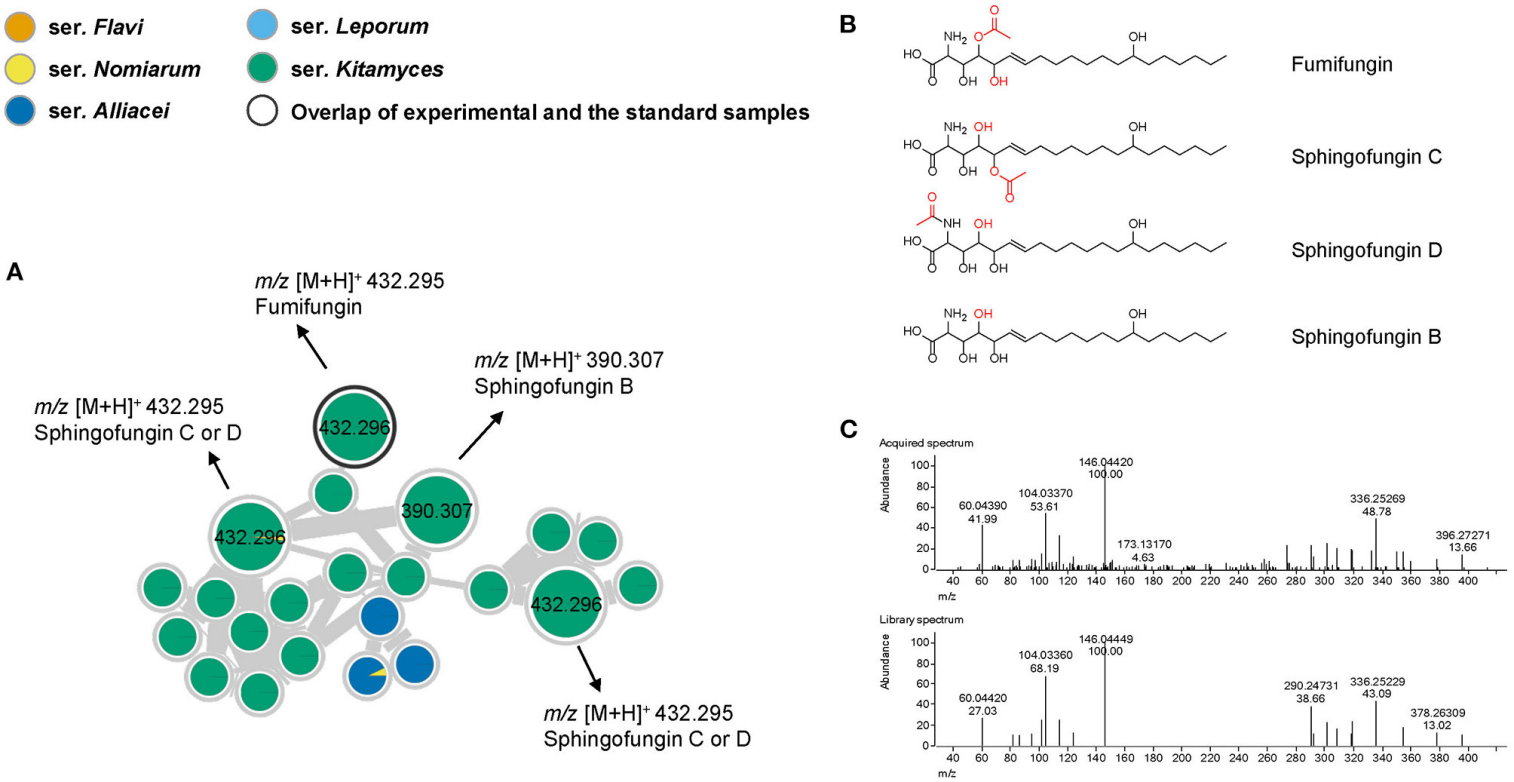
**(A)** Nodes of compounds related to the fumifungin and sphingofungins. **(B)** Structures of identified compounds in this cluster. **(C)** Comparison of MS/MS spectra of fumifungin in acquired spectrum (upper) and library spectrum (lower). Nodes with bold outlined black circles are the overlap of molecules and the standard compounds, the nodes marked with asterisk are the molecules found from the structural relationship shown in molecular networks.

The workflow used here also allowed the rapid identification of a cluster representing analogs of the antifungal agent fumifungin (Figure 6A) (Mukhopadhyay et al., 1987). From previous studies, fumifungin have only been reported from *A. fumigatus* and *A. lentulus* (Larsen et al., 2007), however here we for the first time report fumifungin also from section *Flavi*. Detection of three additional nodes, suggested the presence of several analogs that could be putatively identified as sphingofungin B, C and D based on their elemental composition and similarities in their fragmentation patterns (Figures 6B,C). These metabolites share a similar backbone to the carcinogenic mycotoxins, fumonisins, and may therefore contribute to various serious adverse health outcomes (Frisvad et al., 2009). Fumifungins do not have characteristic or easily recognized UV absorption spectra, making them undetectable solely by liquid-chromatography-diode array detection-mass spectrometry methodology, which is probably why they have been overlooked in other studies. It is worth noting that in this study fumifungin was only produced by all species in series *Kitamyces*, whereas sphingofungin C or D were also detected in the series *Nomiarum* in *A. pseudonomiae* extracts.

Tenuazonic acid and its analog valine-tenuazonic acid were both dereplicated by our in-house database. By checking the producers of this cytotoxic mycotoxin, we identified two producers, *A. arachidicola* and *A. leporis*, which have not previously been described to produce tenuazonic acid.

In addition to the more famous compounds discussed above, the cyclic peptides aspergillicin A and B were detected by the GNPS library. Moreover, the compounds desertorin A and desertorin B were dereplicated in four species by our in-house database and confirmed by fragmentation patterns. Finally, some metabolites that could not be annotated by public databases are likely to be putative novel compounds (Supplementary Figures S1, S2). The MS/MS spectra of all herein mentioned compounds can be found in Supplementary Figures S3—S11.

The overall dereplicated compounds in section *Flavi* and their producers in this study are shown in Table 1. This result shows how the efficient annotation of a molecular network may allow the targeting of unexpected chemistries from previously investigated species. Moreover, this result shows that molecular networking can effectively annotate known compounds for many species in section *Flavi* and discover unexpected compounds from previously studied species.

## Discussion

A comparative genomics study including 23 *Flavi* species have been conducted recently, revealing a high number of secondary metabolite gene clusters and identifying high genome diversity (Kjærbølling et al., 2020). However, in only a few studies have the researchers conducted a comprehensive exploration of the metabolites for the *Flavi* section (Uka et al., 2019). To depict the chemical diversity and distribution of secondary metabolites in 28 *Aspergillus* section *Flavi* extracts, we have shown that a molecular networking-based exploration of extracts of a large number of taxonomically related fungal species can efficiently highlight important mycotoxins and species-specific secondary metabolites. Moreover, this study highlights the chemodiversity and potential for secondary metabolite production within section *Flavi*.

In the previous study, each *Aspergillus* species genome within section *Flavi* was predicted to have an average of 73 secondary metabolite gene clusters, which is more than 15 extra per species compared with species in genus *Penicillium* (Nielsen et al., 2017). In addition, each *Aspergillus* species has an average of 6.8 unique secondary metabolite gene clusters (Kjærbølling et al., 2020). In our study, we annotated an average of 12 know secondary metabolites for each species. In particular, we have identified no more than 10 known compounds from each species in series *Leporum*, indicating there are still considerable potential for discovering novel compounds in species in this series.

This mass spectrometry based strategy allowed rapid identification of the producers of previously known type of compounds, including aflatoxins, ochratoxins, cyclopiazonic acids, aflatrems, tenuazonic acids, miyakamides. However, aflatoxins were not detectable in *A. aflatoxiformans* (IBT 3651) and *A. nomiae* (IBT 5054) under the given growth conditions, despite that these species have previously been reported to produce aflatoxins (Frisvad et al., 2019). One reason might be that different isolates belonging to the same species have different abilities to produce the mycotoxin. The *A. aflatoxiformans* strain we used in this research is the formerly suggested neotype of *Aspergillus parvisclerotigenus* (Frisvad et al., 2019), and this strain may not have the ability to produce aflatoxins on the CYA media even though it has the aflatoxin gene cluster.

Notably, known mycotoxins previously undescribed in less studied species in section *Flavi* were identified here. This included fumifungin and sphingofungin B–D. In addition, we showed that *A. alliaceus* and *A. arachidicola* are new cyclopiazonic acid producers, and that *A. arachidicola* and *A. leporis* are new tenuazonic acid producers. In this study, tenuazonic acid was detected in all of the species in the series *Kitamyces* and only in *A. arachidicola* in the series *Flavi*. This was quite surprising as the recent bioinformatics analysis of genome sequences of the species from section *Flavi* did not indicate a gene cluster related to production of tenuazonic acid for the same strains of *A. caelatus, A. pseudocaelatus and A. arachidicola* as used in this study (Kjærbølling et al., 2020). In addition, the naturally occurring toxins, fumifungins, have previously only been reported from *A. fumigatus* and *A. lentulus* (Frisvad et al., 2009). The corresponding biosynthetic gene cluster has not yet been annotated from the species in section *Flavi*. By using molecular networking mapped with the taxonomic information, these series-specific compounds were quickly identified from species within the *Flavi* section. The above shows that although this section has been the subject of extensive studies at the genetic level, research at the chemical level could provide insights for some unannotated gene clusters.

In their recent study Kjærbølling and co-workers found gene clusters similar to those described for naphthopyrone, nidulanin A, azanigerone in most *Flavi* genomes (Kjærbølling et al., 2020), however none of these compounds were detected here, potentially because we only inoculated the fungi on CYA medium, or because only variants of these compounds are produced by fungi in section *Flavi*. In general we have good experiences with this substrate leading to production of a broad spectrum of different types of secondary metabolites. However, the use of additional media, following the “One strain many compounds” (OSMAC) approach (Bode et al., 2002; Hewage et al., 2014), would very likely have led to expression of additional biosynthetic pathways.

To investigate the chemodiversity of species in section *Flavi*, we applied the classical molecular networking from GNPS web platform in conjunction with an in-house database (Kildgaard et al., 2014), which efficiently showed the chemical profile from many species together and at the same time increased the confidence of molecular identifications because of the implementation of MS/MS for providing structure information. However, this approach also has several limitations. The first is that the number of nodes in the network does not correspond exactly to the number of metabolites, as different adducts or different charge states of the same chemical species can generate different nodes. Rather, molecular networking provides an overview of the different chemistries detected by mass spectrometry. Secondly, the classical molecular networking doesn't incorporate the retention time from chromatographic separation so that it might not distinguish structural isomers very well. Thirdly, some compounds are not easily fragmented, which means that molecular networking solely cannot identify them and show the relationship between these compounds very well. In addition to the classical molecular networking, the GNPS platform has another method for the generation of molecular networks, called feature-based molecular networking, which needs an alignment tool, such as MZmine (Pluskal et al., 2010), to process the LC-MS/MS data before uploading to the GNPS platform. By incorporating MS^1^ information, such as isotope patterns and retention times, feature-based molecular networking enables quantitative analysis, which allow the downstream metabolomics statistical analysis, and allow for distinguishment of isomers producing similar MS/MS spectra.

The classical molecular networking method is based on the MS-Cluster algorithm, which can align unknown molecules with similar fragmentation data rapidly. A recent study pointed out the limitations, emphasizing that despite the fact that cosine-based methods are very good at revealing nearly equal spectra, they are not as well-suited to handle molecules with multiple local chemical modifications (Huber et al., 2021). They introduced Spec2Vec, a novel spectral similarity score inspired by a natural language processing algorithm, Word2Vec that can be adapted to learn meaningful relations between mass fragments and neutral losses in mass fragmentation spectra (Mikolov et al., 2013; Huber et al., 2021). One limitation of Spec2Vec as compared to cosine scores is that it needs training data to learn the fragment peak relationships. In addition, a recent study showed that 2D-NMR-based untargeted metabolomic analysis is also a promising tool for dereplication of known compounds from complex natural products mixtures. Future research could try different strains from one species, getting more information by using different algorithms (Spec2Vec score and cosine score) and analysis tools, such as NMR based analysis and feature-based molecular networking (Egan et al., 2020; Nothias et al., 2020), to show more comprehensive relationships between many species and discovering more metabolites in complex mixtures.

Altogether, the analyses revealed several potentially novel compounds and predicted putative structures based on fragmentation patterns, which has initiated new projects in our group. In addition, our results have important reference value for future research on species in the *Flavi* section because they show all the producers of those bioactive metabolites and may avoid repeated isolation to a certain extent. Future investigations of secondary metabolite production in section *Flavi* will likely lead to even further new insights into their chemical diversity.

## Data Availability Statement

The datasets presented in this study can be found in online repositories. The names of the repository/repositories and accession number(s) can be found below: https://gnps.ucsd.edu/ProteoSAFe/status.jsp?task=1479da25db9a49b79bbfe4cfc7084e51. MASSIVE: doi: 10.25345/C5GZ1W.

## Author Contributions

XW, KS, JF and TL designed the experiments. SK performed culturing, extraction and the dereplication by in-house database. XW and KS performed the dereplication by in-house database and the molecular networking analysis. XW wrote the paper with contribution from all authors. All authors contributed to the article and approved the submitted version.

## Conflict of Interest

The authors declare that the research was conducted in the absence of any commercial or financial relationships that could be construed as a potential conflict of interest. The handling Editor declared a past co-authorship with one of the authors JF.

## Publisher's Note

All claims expressed in this article are solely those of the authors and do not necessarily represent those of their affiliated organizations, or those of the publisher, the editors and the reviewers. Any product that may be evaluated in this article, or claim that may be made by its manufacturer, is not guaranteed or endorsed by the publisher.

## Footnotes

^1^https://www-reaxys-com (accessed February 17, 2017).
