# Supplementary material for: Mass Spectrometry-Based Network Analysis Reveals New Insights Into the Chemodiversity of 28 Species in Aspergillus section Flavi
Source: Front Fungal Biol. 2021 Aug 11;2:719420. doi: 10.3389/ffunb.2021.719420 (PMC10512371; doi:10.3389/ffunb.2021.719420)
Supplement: Supplementary file 1 [file Data_Sheet_1.docx]

**Supplementary Information**

**Table of contents**

**Supplementary Table S1**: Overview of the fungal strains used in this study.

**Supplementary** **Table S2:** The overall dereplicated compounds in this study.

**Supplementary** **Figure S1:** Cluster F represents the nodes of detected miyakamides.

**Supplementary** **Figure S2:** The nodes with dark red outline represent the species-specific metabolites, which are only produced by *Aspergillus* *aspearensis*.

**Supplementary** **Figure S3:** High resolution MS/MS spectra of aflatoxins at 20 eV.

**Supplementary** **Figure S4:** High resolution MS/MS spectra of ochratoxins at 20 eV.

**Supplementary** **Figure S5:** High resolution MS/MS spectra of cyclopiazonic acids at 20 eV.

**Supplementary Figure S6:** High resolution MS/MS spectra of aflatrems and aflavinines at 20 eV.

**Supplementary Figure S7:** High resolution MS/MS spectra of fumifungins at 20 eV.

**Supplementary** **Figure S8:** High resolution MS/MS spectra of tenuazonic acids at 20 eV.

**Supplementary** **Figure S9:** High resolution MS/MS spectra of aspergillicins at 40 eV.

**Supplementary** **Figure S10:** High resolution MS/MS spectra of miyakamides at 20 eV.

**Supplementary** **Figure S11:** High resolution MS/MS spectra of some representative metabolites at 20 eV.

**Table S1**. Overview of the fungal strains used in this study.

| Series | Species | Isolate number |
| --- | --- | --- |
| *Flavi* | *Aspergillus sojae* | IBT 21650 |
| *Flavi* | *A.pipericola* | IBT 24628 |
| *Flavi* | *A.oryzae* | IBT 28103 |
| *Flavi* | *A.flavus* | IBT 29624 |
| *Flavi* | *A.cerealis* | IBT 32067 |
| *Flavi* | *A.austwickii* | IBT 32076 |
| *Flavi* | *A.subflavus* | IBT 33354 |
| *Flavi* | *A.aflatoxiformans* | IBT 3651 |
| *Flavi* | *A.parasiticus* | IBT 27186 |
| *Flavi* | *A.arachidicola* | IBT 27190 |
| *Flavi* | *A.minisclerotigenes* | IBT 27196 |
| *Flavi* | *A.sergii* | IBT 32293 |
| *Flavi* | *A.transmontanensis* | IBT 32313 |
| *Flavi* | *A.novoparasiticus* | IBT 32314 |
| *Kitamyces* | *A.pseudotamarii* | IBT 21090 |
| *Kitamyces* | *A.caelatus* | IBT 21091 |
| *Kitamyces* | *A.tamarii* | IBT 27182 |
| *Kitamyces* | *A.pseudocaelatus* | IBT 27191 |
| *Nomiarum* | *A.pseudonomius* | IBT 27864 |
| *Nomiarum* | *A.nomius* | IBT 5054 |
| *Nomiarum* | *A.luteovirescens* | IBT 23536 |
| *Alliacei* | *A.alliaceus* | IBT 13376 |
| *Alliacei* | *A.vandermerwei* | IBT 13865 |
| *Alliacei* | *A.albertensis* | IBT 14317 |
| *Alliacei* | *A.neoalliaceus* | IBT 33356 |
| *Leporum* | *A.leporis* | IBT 3609 |
| *Leporum* | *A.aspearensis* | IBT 34544 |
| *Leporum* | *A.hancockii* | IBT 35031 |

**Table S2**. The overall dereplicated compounds in this study. The compounds highlighted in green color are dereplicated by the GNPS library. The compounds marked with asterisk are found by the in-house library reseach; and the compounds highlight in yellow are the important mycotoxins for which standards have been used for generation of reference spectra for molecular network analysis.

| **Compounds number** | **Network clusters** | **Compounds name** | **Molecular Formula** | **m/z** | **Mass (MFG^*^)** |
| --- | --- | --- | --- | --- | --- |
| 1 | Aflatoxins | **aflatoxin B1*** | C_17_H_12_O_6_ | 313.0707 | 312.0634 |
| 2 |  | **aflatoxin B3*** | C_16_H_14_O_6_ | 303.0864 | 302.0791 |
| 3 |  | **aflatoxin G1*** | C_17_O_7_H_12_ | 329.0655 | 328.0586 |
| 4 |  | **aflatoxin GM2** | C_17_O_8_H_14_ | 347.0762 | 346.0689 |
| 5 |  | **O-methylsterigmatocystin** | C_19_O_6_H_14_ | 339.0872 | 338.079 |
| 6 |  | **Dihydroaflatoxicol** | C_16_O_7_H_12_ | 317.0657 | 316.0583 |
| 7 | Ochratoxins | **Ochratoxin A*** | C_20_H_18_ClNO_6_ | 404.0898 | 403.0826 |
| 8 |  | **Ochratoxin B*** | C_20_NO_6_H_19_ | 370.1291 | 369.1218 |
| 9 |  | **Ochratoxin beta** | C_11_H_10_O_5_ | 223.0605 | 222.0528 |
| 10 |  | **CTKSE9855** | C_20_H_19_NO_7_ | 386.1239 | 385.1162 |
| 11 | Cyclopiazonic acids | **Cyclopiazonic acid *** | C_20_N_2_O_3_H_20_ | 337.1552 | 336.148 |
| 12 |  | **Speradine A*** | C_21_N_2_O_4_H_22_ | 367.1656 | 366.1585 |
| 13 |  | **2-oxo-cyclopiazonic acid** | C_20_N_2_O_4_H_20_ | 353.1502 | 352.1423 |
| 14 |  | **3-OH-speradine A** | C_21_N_2_O_5_H_22_ | 383.161 | 382.1529 |
| 15 |  | **speradine F** | C_22_N_2_O_6_H_26_ | 415.186 | 414.1791 |
| 16 | Fumifungins | **Fumifungin*** | C_22_H_41_NO_7_ | 432.2953 | 431.288 |
| 17 |  | **Sphingofungin B** | C_20_H_39_NO_6_ | 390.2854 | 389.2777 |
| 18 |  | **Sphingofungin C** | C_22_H_41_NO_7_ | 432.2965 | 431.2883 |
| 19 |  | **Sphingofungin D** | C_22_H_41_NO_7_ | 432.2969 | 431.2883 |
| 20 | Aflatrems | **Aflatrem*** | C_32_NO_4_H_39_ | 502.2956 | 501.2881 |
| 21 |  | **Paspalinine*** | C_27_NO_4_H_31_ | 434.2328 | 433.2255 |
| 22 |  | **Paspaline** | C_28_NO_2_H_39_ | 422.3057 | 421.2981 |
| 23 |  | **Hydroxypaspalinine** | C_27_H_31_NO_5_ | 450.2272 | 449.2202 |
| 24 |  | **Penerpenes** | C_27_H_31_NO_5_ | 450.2276 | 449.2202 |
| 25 |  | **Asperindoles** | C_29_NO_6_H_33_ | 492.2390 | 491.2321 |
| 26 | Aflavinines | **14-hydroxyaflavinine** | C_28_NO_2_H_39_ | 422.3051 | 421.2981 |
| 27 |  | **Aflavinine** | C_28_NOH_39_ | 406.3098 | 405.3032 |
| 28 |  | **Dihydroxyaflavinine** | C_28_NO_3_H_39_ | 438.3004 | 437.2930 |
| 29 | Tenuazonic acids | **Tenuazonic acid*** | C_10_NO_3_H_15_ | 198.1128 | 197.1055 |
| 30 |  | **Valine-tenuazonic acid*** | C_9_H_13_NO_3_ | 184.0964 | 183.0891 |
| 31 | Aspergillicins | **Aspergillicin A** | C_38_N_6_O_9_H_56_ | 763.4004 | 740.4109 |
| 32 |  | **Aspergillicin B** | C_37_N_6_O_9_H_54_ | 749.384 | 726.3952 |
| 33 |  | **Aspergillicin C** | C_37_N_6_O_8_H_54_ | 733.3905 | 710.4003 |
| 34 |  | **Aspergillicin G** | C_38_N_6_O_8_H_56_ | 747.4066 | 724.416 |
| 35 |  | **Aspergillicin E/F** | C_39_N_6_O_9_H_58_ | 777.4161 | 754.4265 |
| 36 | Miyakamides | **Miyakamide A1*/A2*** | C_31_N_4_O_3_H_32_ | 509.2551 | 508.2482 |
| 37 |  | **Miyakamide B1*/B2** | C_31_N_4_O_4_H_32_ | 525.2497 | 524.2431 |
| 38 |  | **Miyakamide analogues with Valine** | C_27_N_4_O_3_H_32_ | 461.2552 | 460.2474 |
| 39 |  | **aspergillamide A/B** | C_28_H_34_N_4_O_3_ | 497.2533 | 474.2631 |
| 40 |  | **Oryzamide A1/A2** | C_28_N_4_O_4_H_34_ | 513.2481 | 490.258 |
| 41 |  | **Ditryptophenaline*** | C_42_N_6_O_4_H_40_ | 693.3192 | 692.3117 |
| 42 |  | **aspergillic acid*** | C_12_N_2_O_2_H_20_ | 225.16 | 224.1527 |
| 43 |  | **desertorin A*** | C_22_H_18_O_8_ | 411.1075 | 410.1002 |
| 44 |  | **desertorin B** | C_23_H_20_O_8_ | 425.1238 | 424.1158 |
| 45 |  | **Ergokonin B*** | C_28_H_42_O_5_ | 481.2924 | 458.3032 |
| 46 |  | **13-Dehydroxypaxilline*** | C_27_H_33_NO_3_ | 420.2532 | 419.2459 |
| 47 |  | **Kojic acid*** | C_6_O_4_H_6_ | 143.0338 | 142.0265 |
| 48 |  | **Phytosphingosine*** | C_18_H_39_NO_3_ | 318.3001 | 317.2928 |
| 49 |  | **(-)-Canadensolide*** | C_11_O_4_H_14_ | 211.0966 | 210.0892 |
| 50 |  | **Chrysogine*** | C_10_N_2_O_2_H_10_ | 191.0816 | 190.0743 |
| 51 |  | **Parasiticolide A*** | C_26_O_8_H_30_ | 493.1838 | 470.1946 |
| 52 |  | **ustilaginoidin C*** | C_28_O_12_H_18_ | 547.0878 | 546.0804 |
| 53 |  | **Sterigmatocystin** | C_18_H_12_O_6_ | 325.0707 | 324.0623 |
| 54 |  | **Lovastatin / mevinolin** | C_24_H_36_O_5_ | 427.2460 | 404.2563 |
| 55 |  | **Lovastatin acid** | C_24_H_38_O_6_ | 445.2563 | 422.2668 |
| 56 |  | **Lovastatin analogue** | C_24_H_40_O_6_ | 447.2723 | 424.2825 |
| 57 |  | **TMC-95A** | C_33_N_6_O_10_H_38_ | 679.2733 | 678.2649 |
| 58 |  | **Deacetylparasiticolide A** | C_24_H_28_O_7_ | 429.1910 | 428.1835 |
| 59 |  | **Heptelidic acid** | C_15_O_5_H_20_ | 281.1387 | 280.1311 |
| 60 |  | **Flavacol** | C_12_N_2_OH_21_ | 209.1650 | 208.1576 |

^*^This column shows that the results was found by the Generate Formulas algorithm (MGF) in MassHunter.


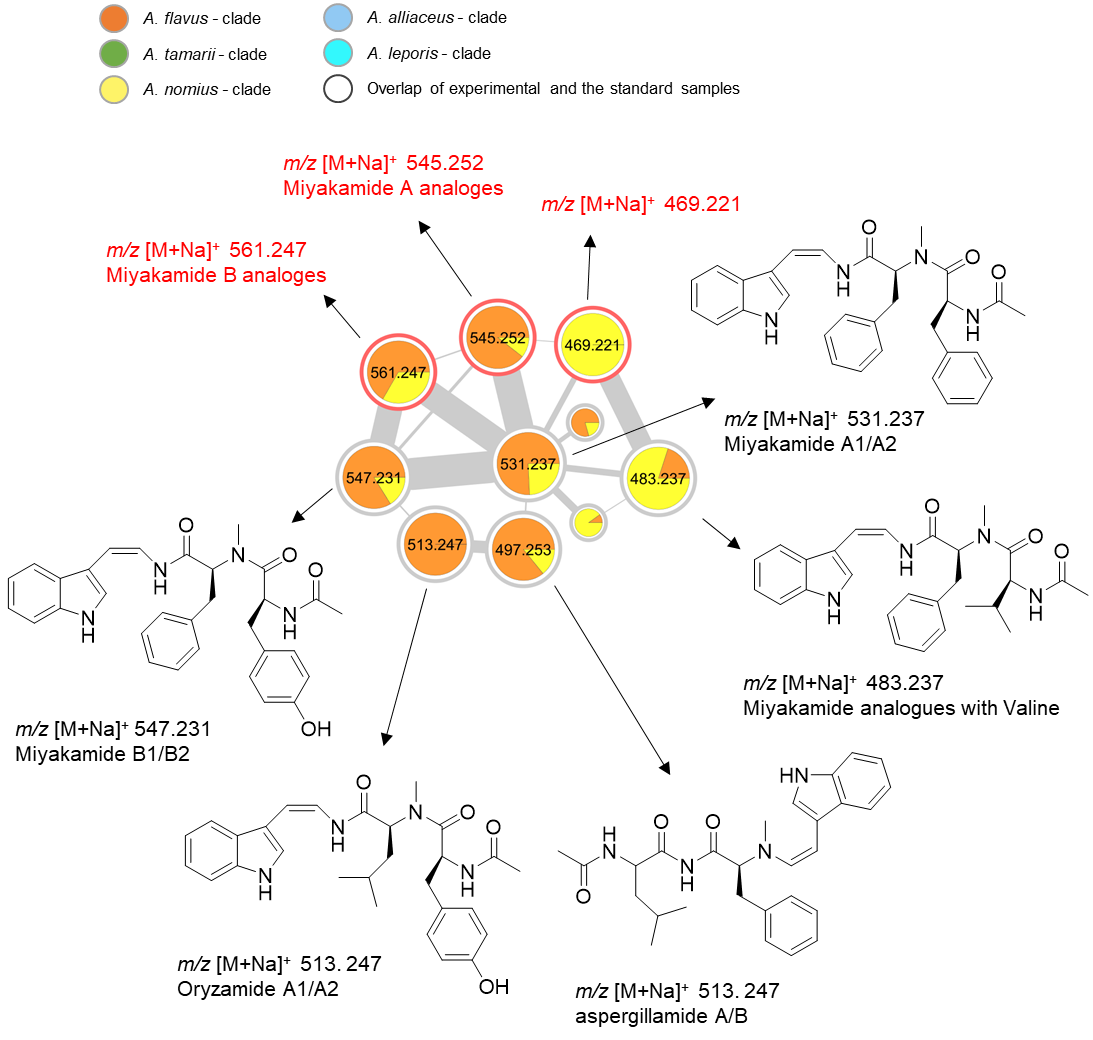


**F**

**Figure S1**. Cluster F represents the nodes of detected miyakamides. The nodes with red outline represent the potentially novel compounds.

In the cluster F (**Figure S1**), Miyakamides were dereplicated manually. They were first isolated from the cultured broth of *Aspergillus flavus* strain and showed inhibitory activity against brine shrimp**.** The map of molecular network shows that Miyakamide A1/A2 is produced by *A. sojae, A. nomius, A. parasitiicus, A. novoparasiticus* and *A. transmontanensis*, and that Miyakamide B1/B2 is produced by *A. sojae, A. nomius, A. novoparasiticus* and *A. transmontanensis*. In addition, the node of miyakamide A (m/z 531) is connected to the precursor mass of m/z 483 with the cosine score of 0.74, a difference of 48 amu less than miyakamide A, suggests the presence of a valine fragment instead of a phenylalanine fragment in the molecule. This is confirmed by checking fragmentation patterns of both compounds. Moreover, Oryzamide A1/A2 and aspergillamide A/B were also identified in MN.


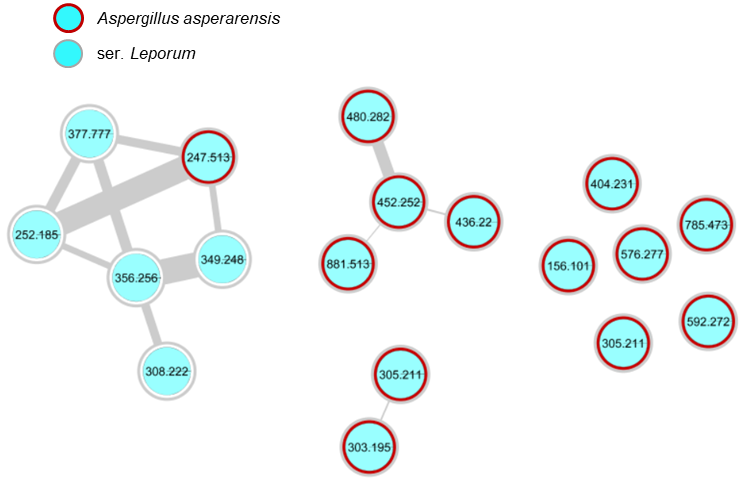


**Figure S2**. The nodes with dark red outline represent the species-specific metabolites, which are only produced by *Aspergillus aspearensis*; the nodes with gray outline represent the series-specific metabolites, which are only produced by series *Leporum*.


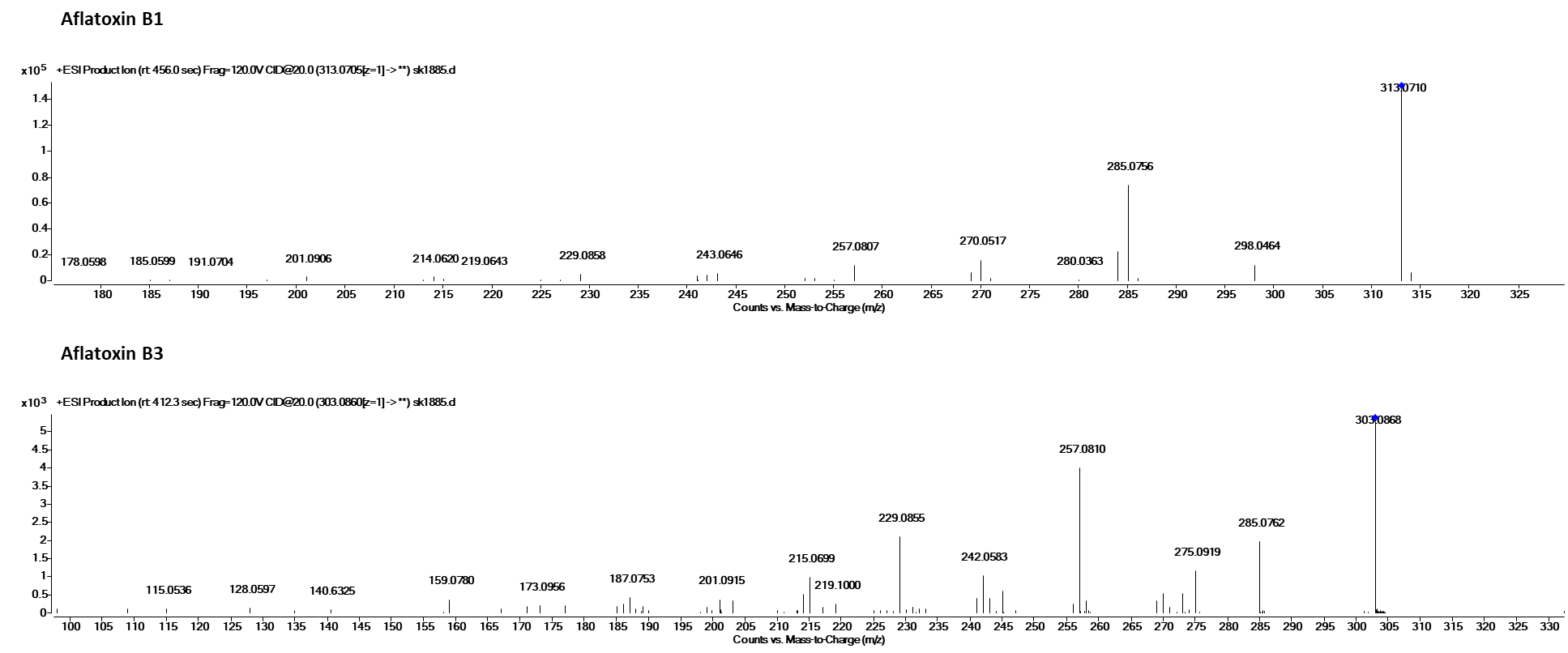


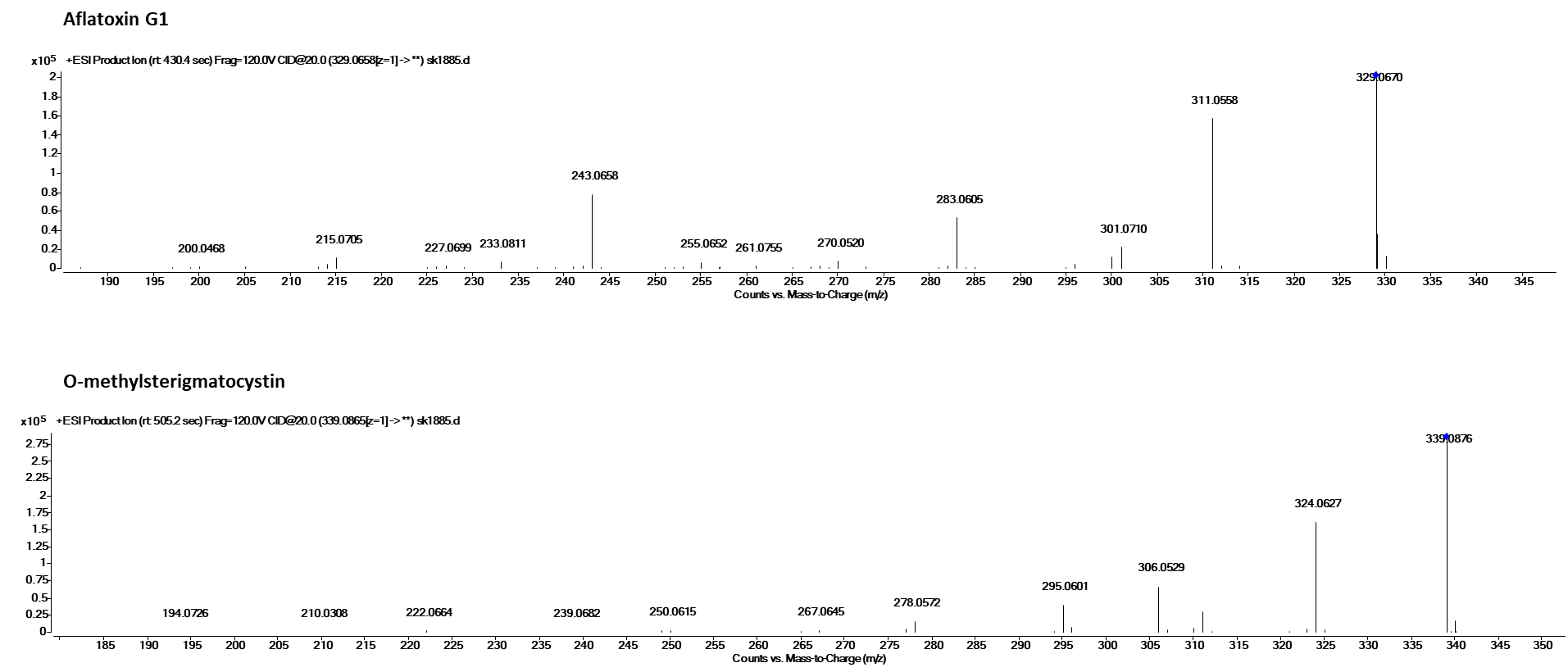


**Figure S3.** High resolution MS/MS spectra of aflatoxins at 20 eV.


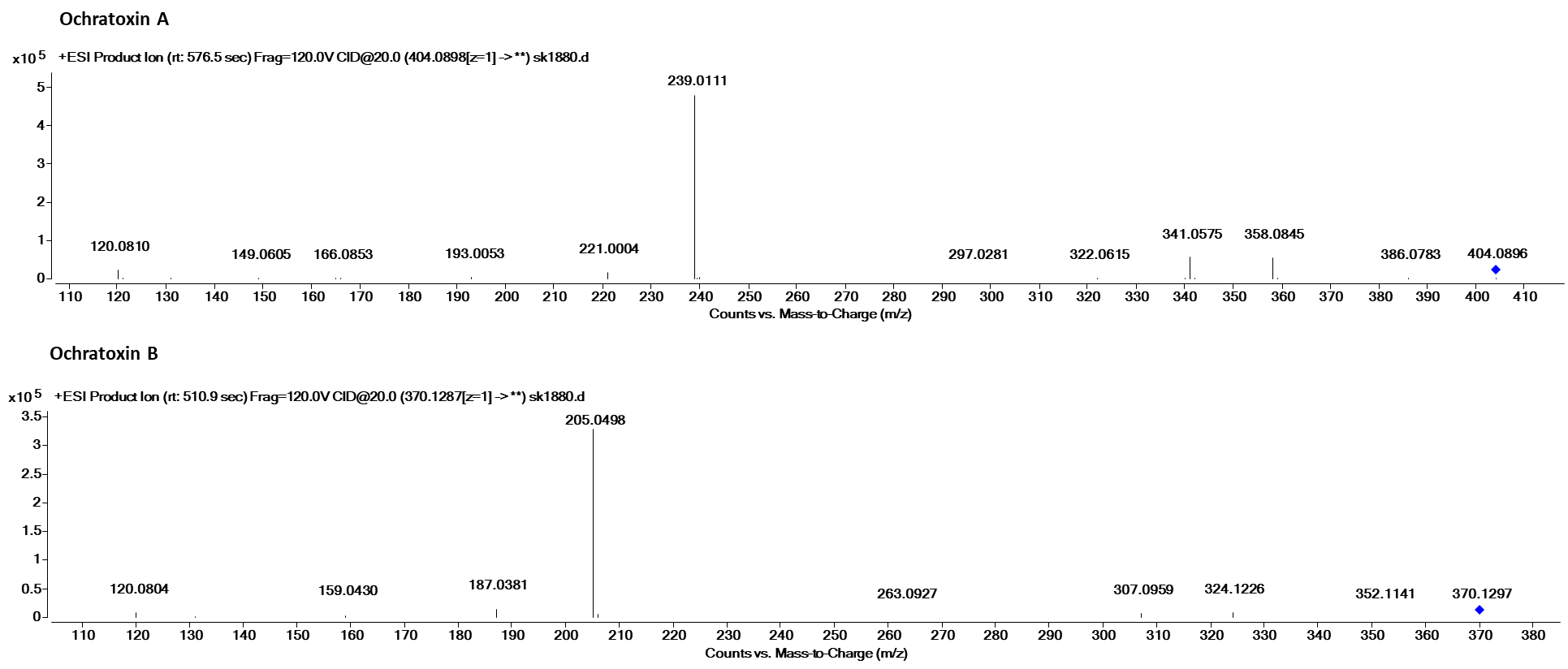


**Figure S4.** High resolution MS/MS spectra of ochratoxins at 20 eV.


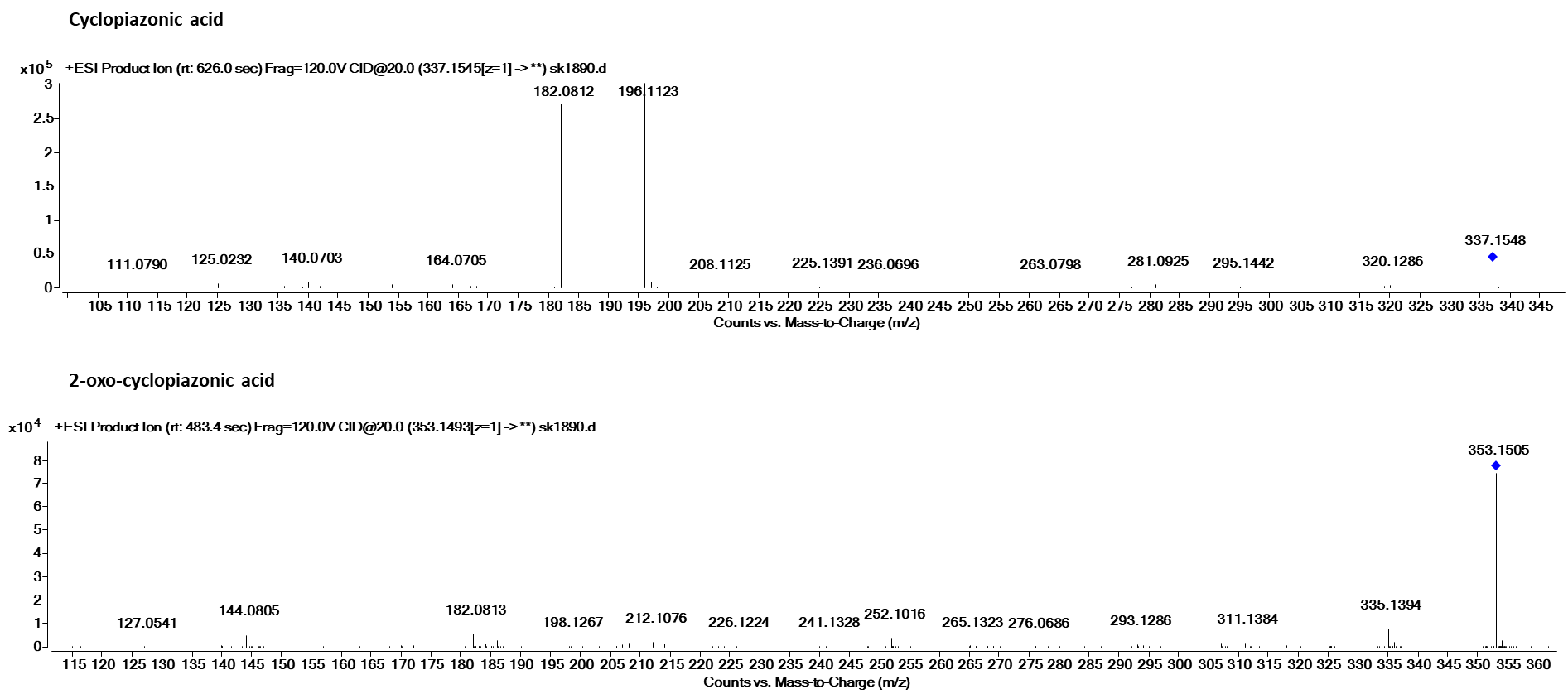


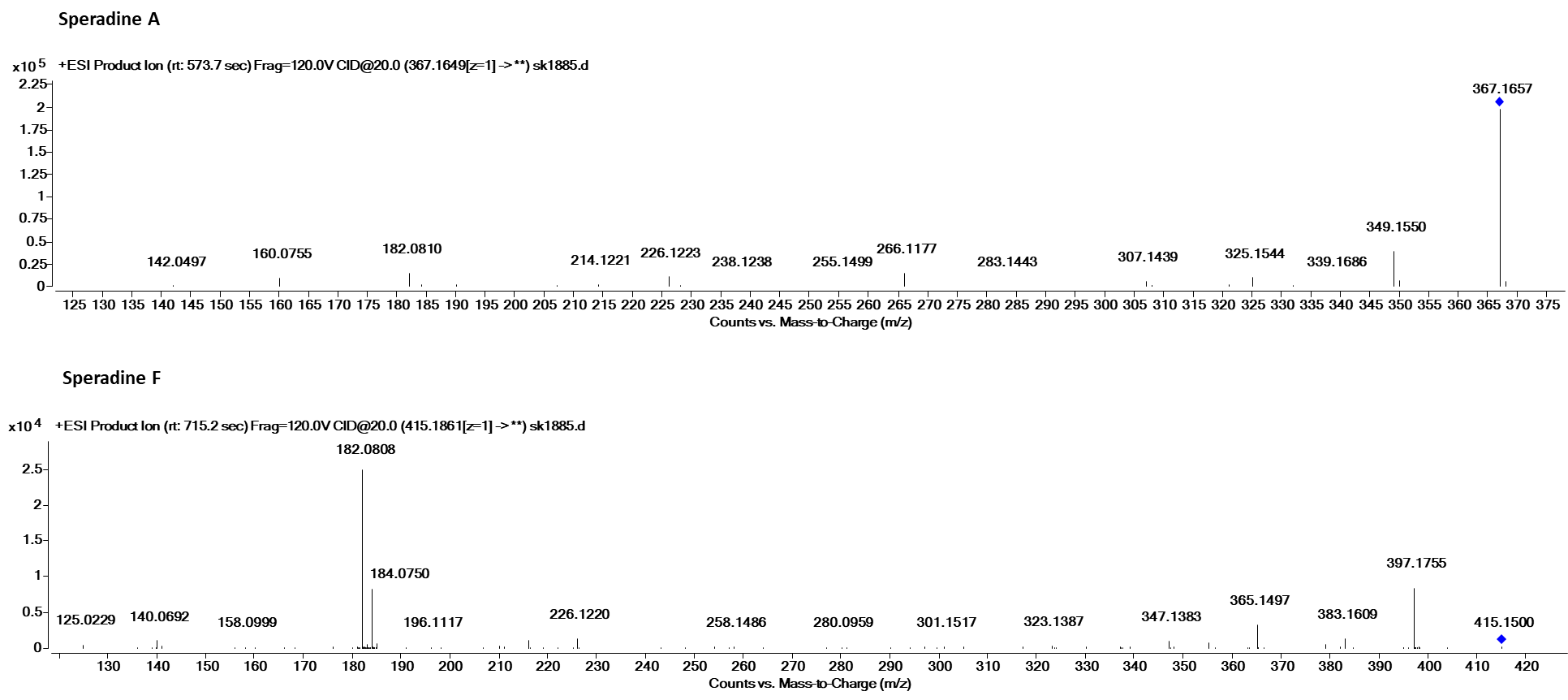


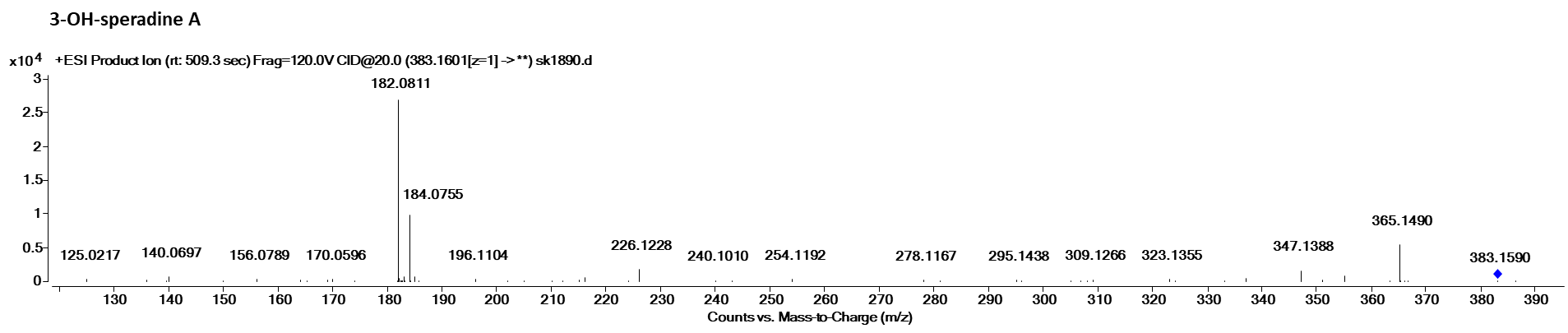


**Figure S5.** High resolution MS/MS spectra of cyclopiazonic acids at 20 eV.


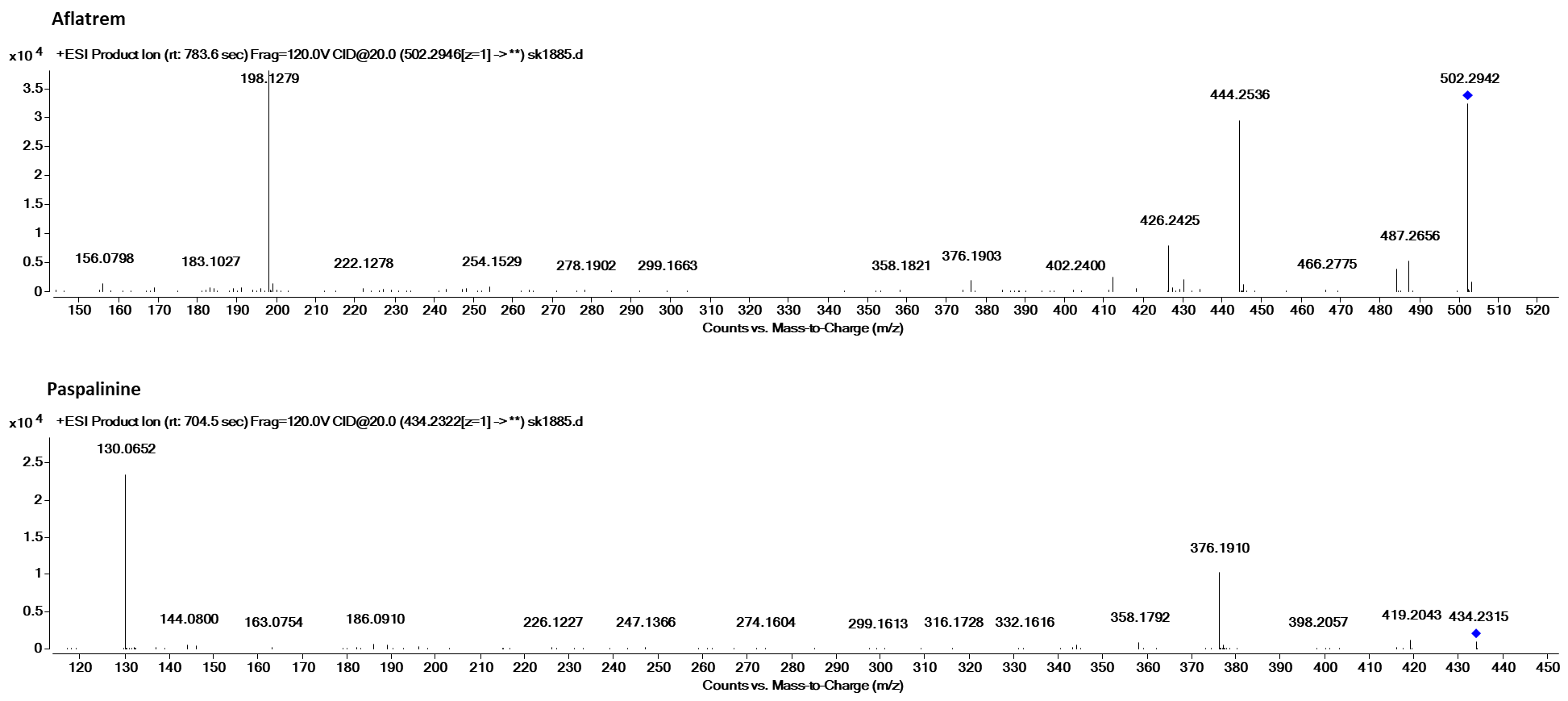


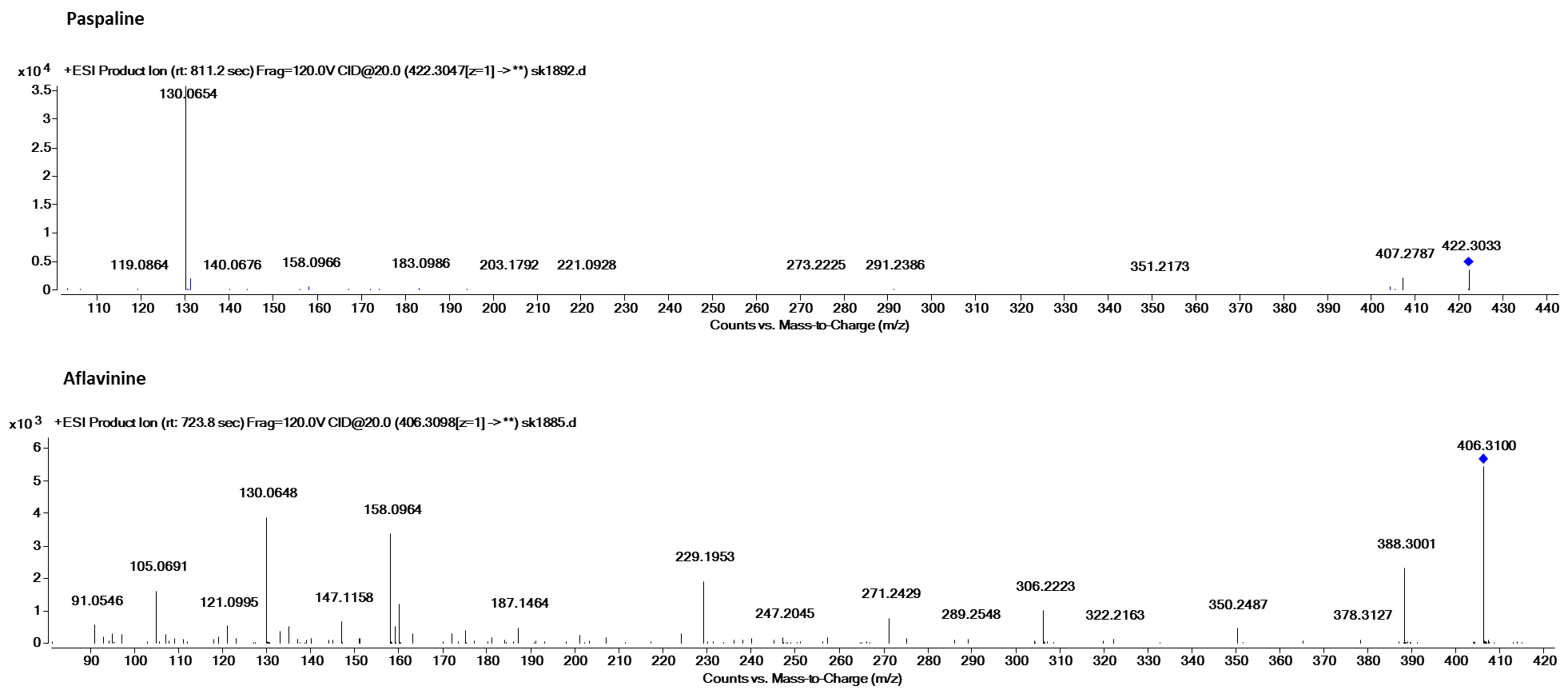


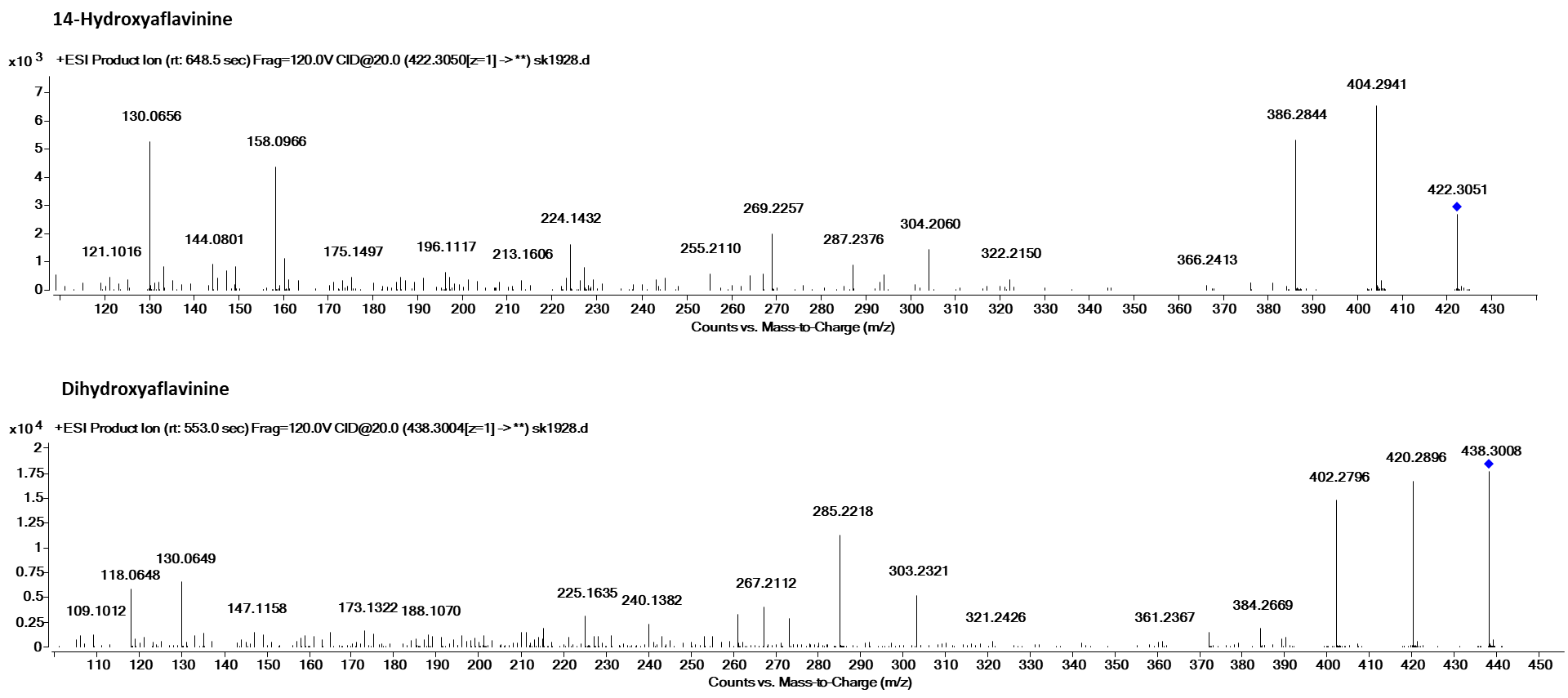


**Figure S6.** High resolution MS/MS spectra of aflatrems and aflavinines at 20 eV.


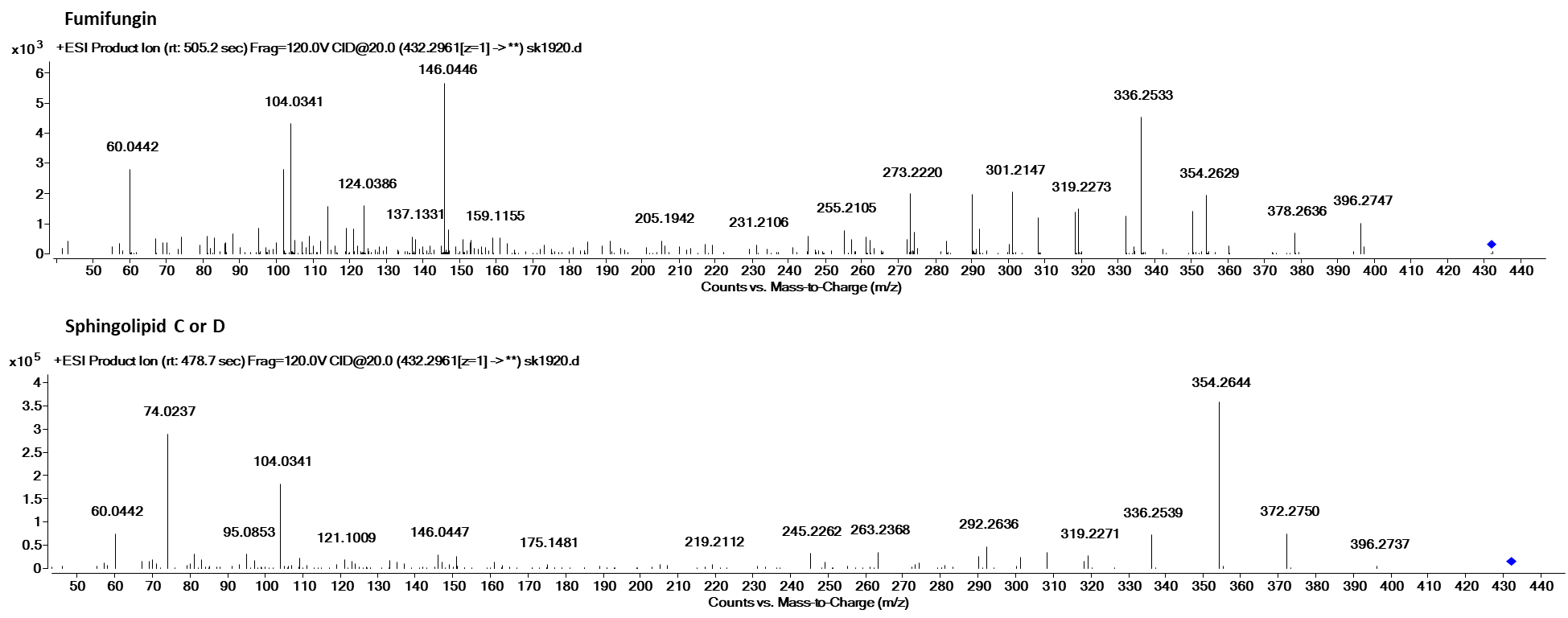


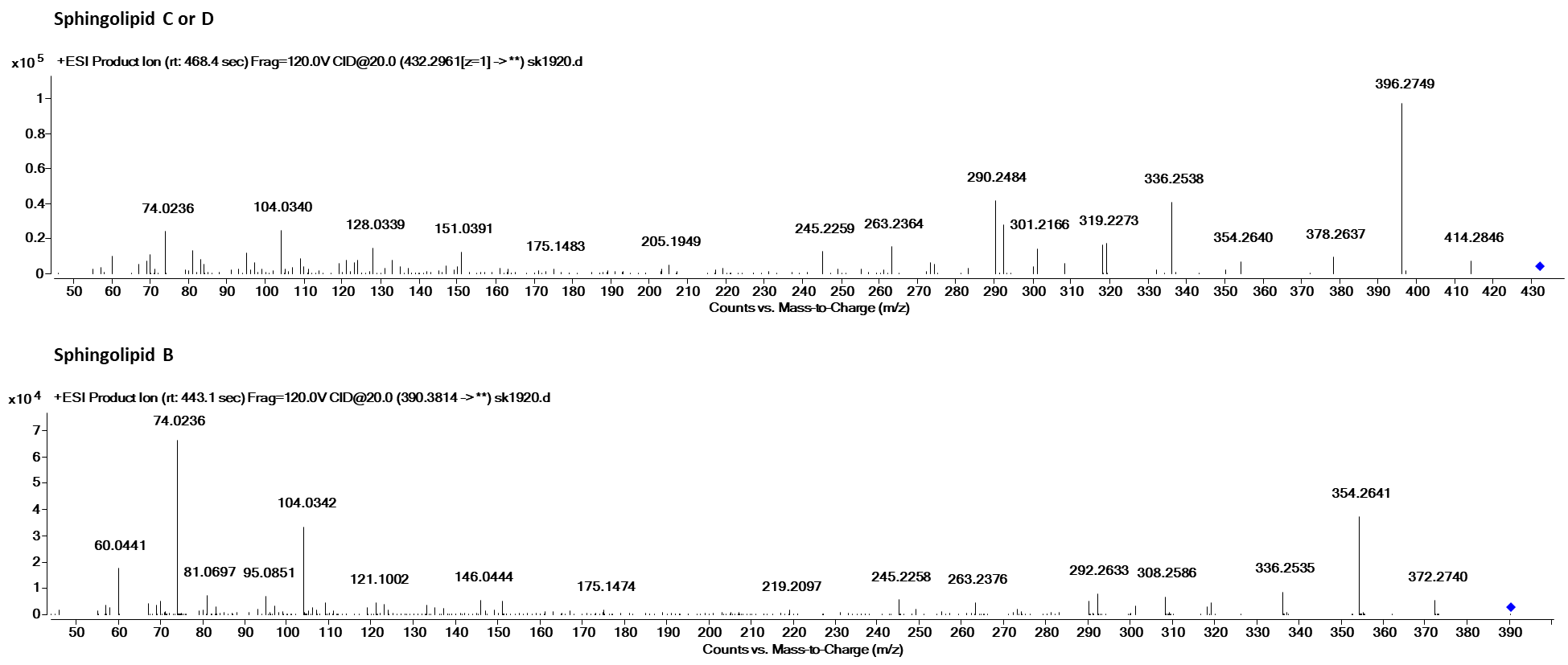


**Figure S7.** High resolution MS/MS spectra of fumifungins at 20 eV.


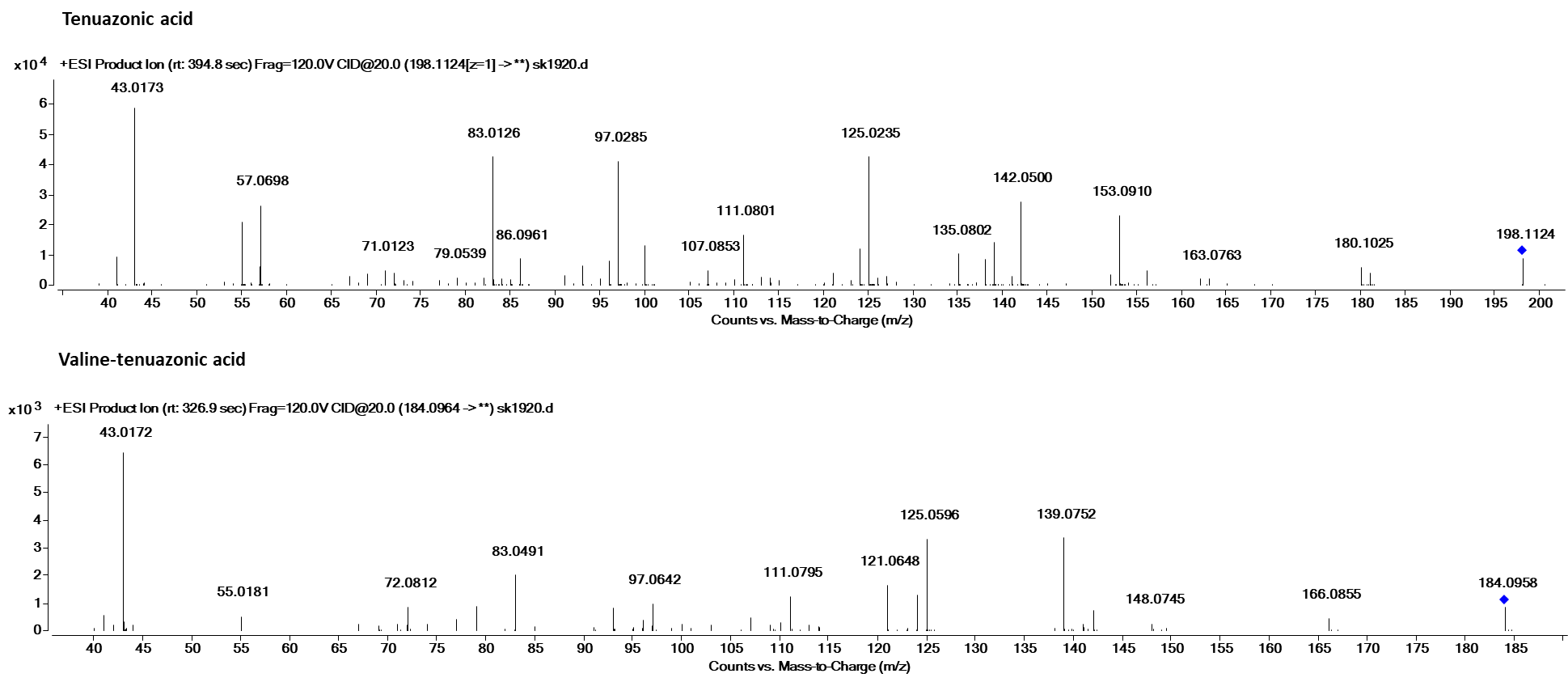


**Figure S8.** High resolution MS/MS spectra of tenuazonic acids at 20 eV.


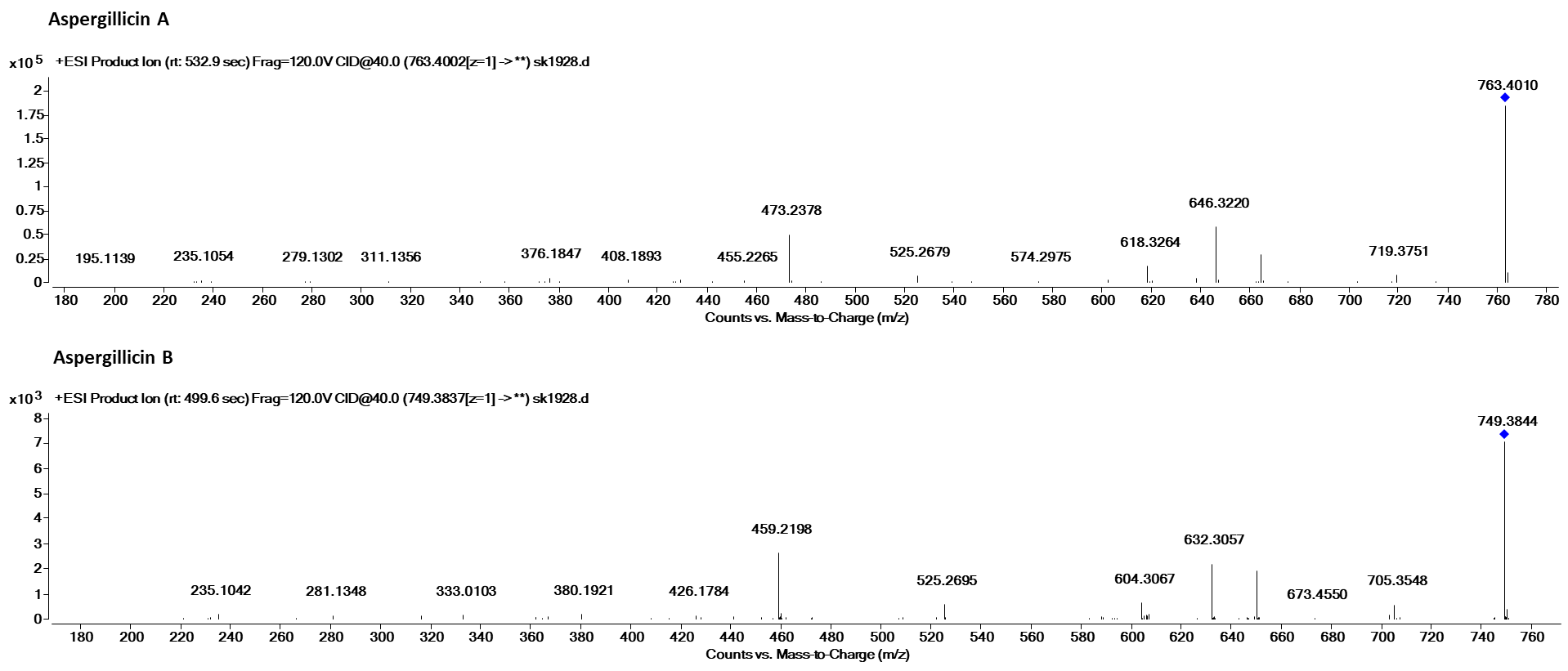


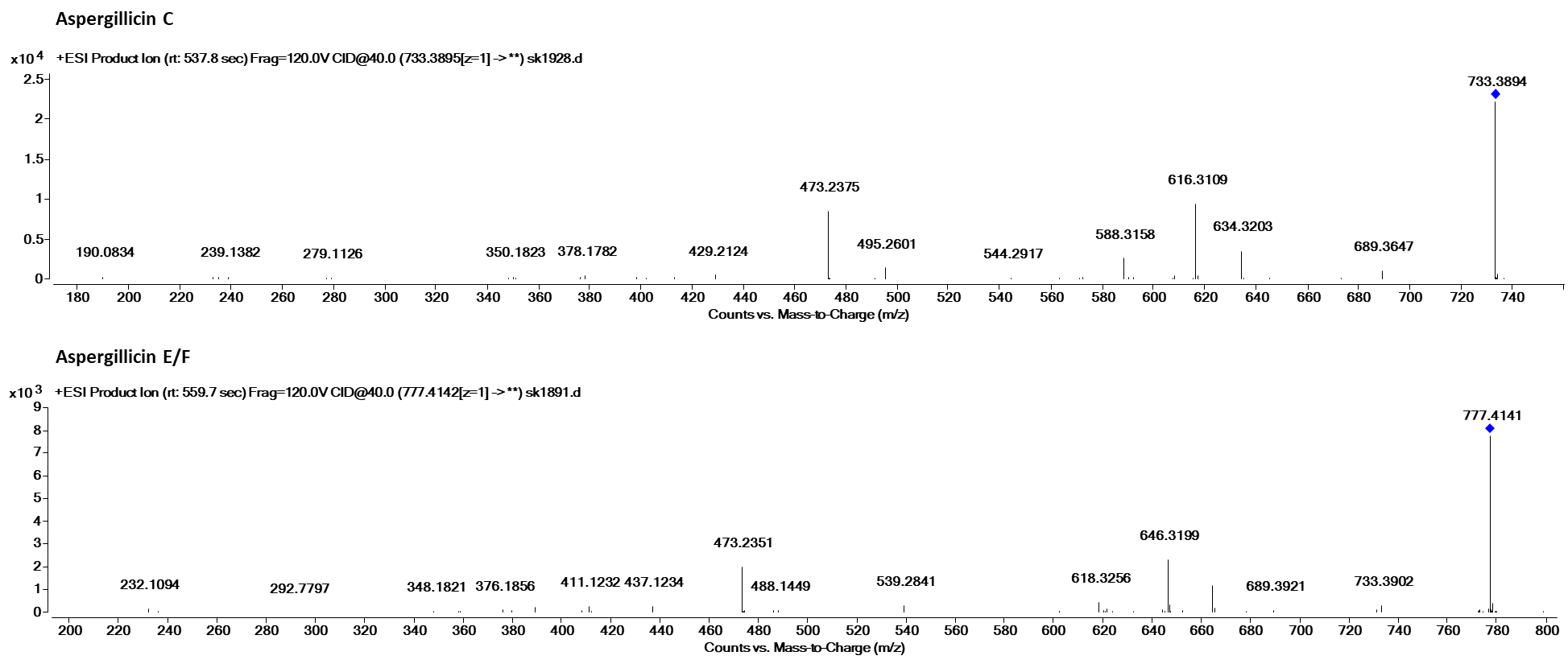


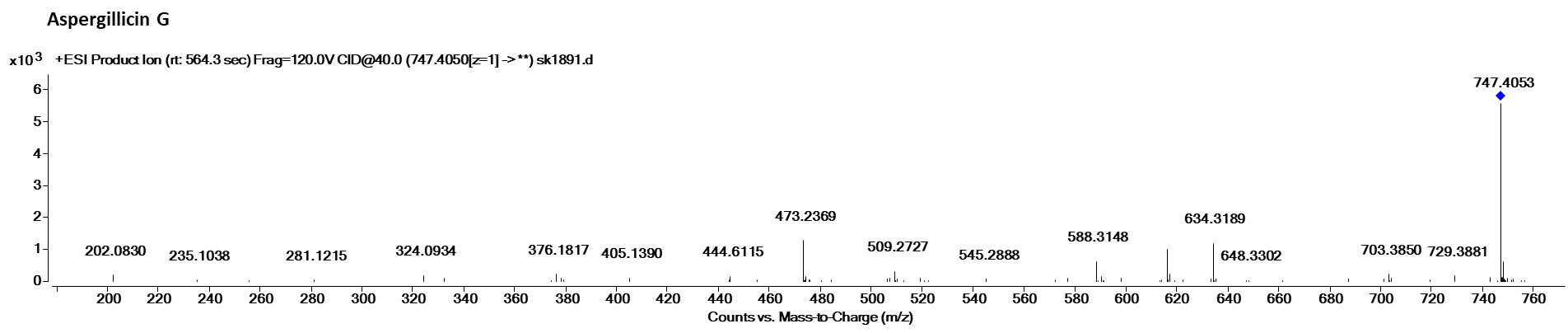


**Figure S9.** High resolution MS/MS spectra of aspergillicins at 40 eV.


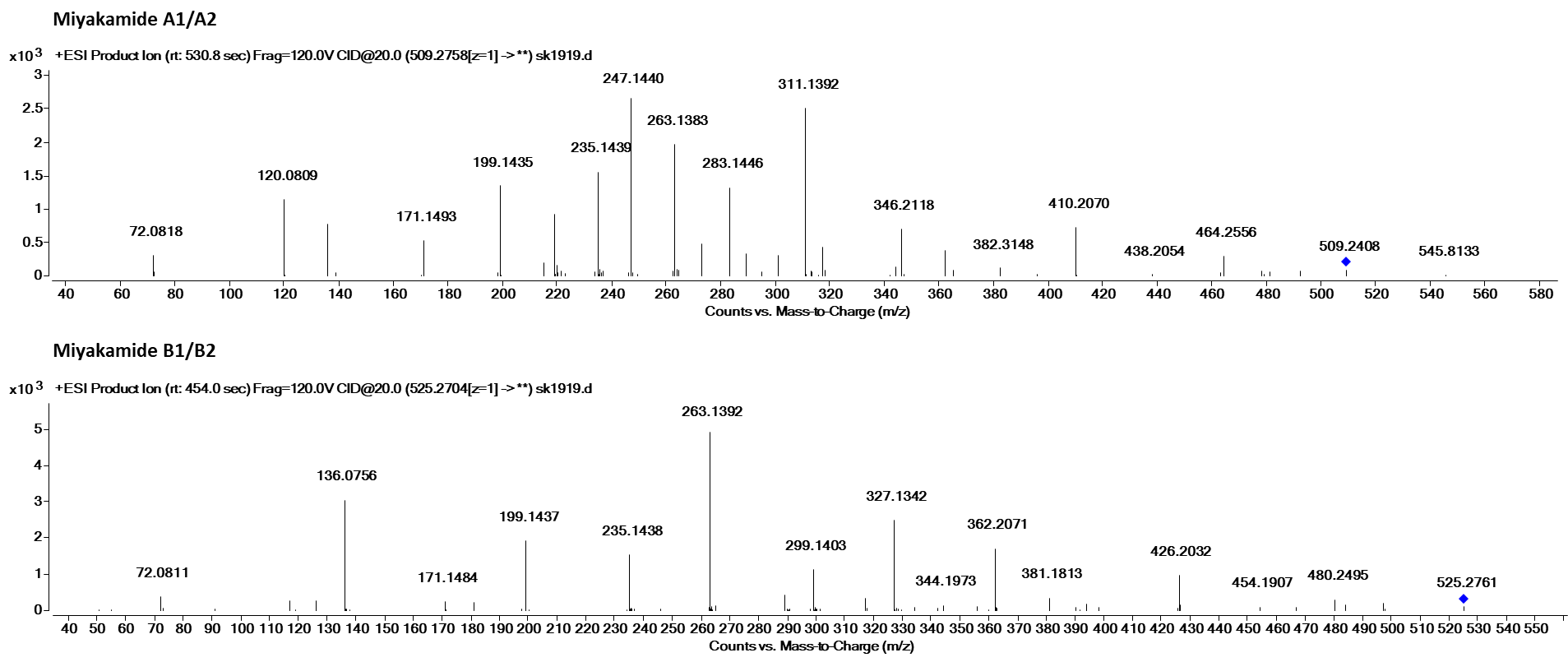


**Figure S10.** High resolution MS/MS spectra of miyakamides at 20 eV.


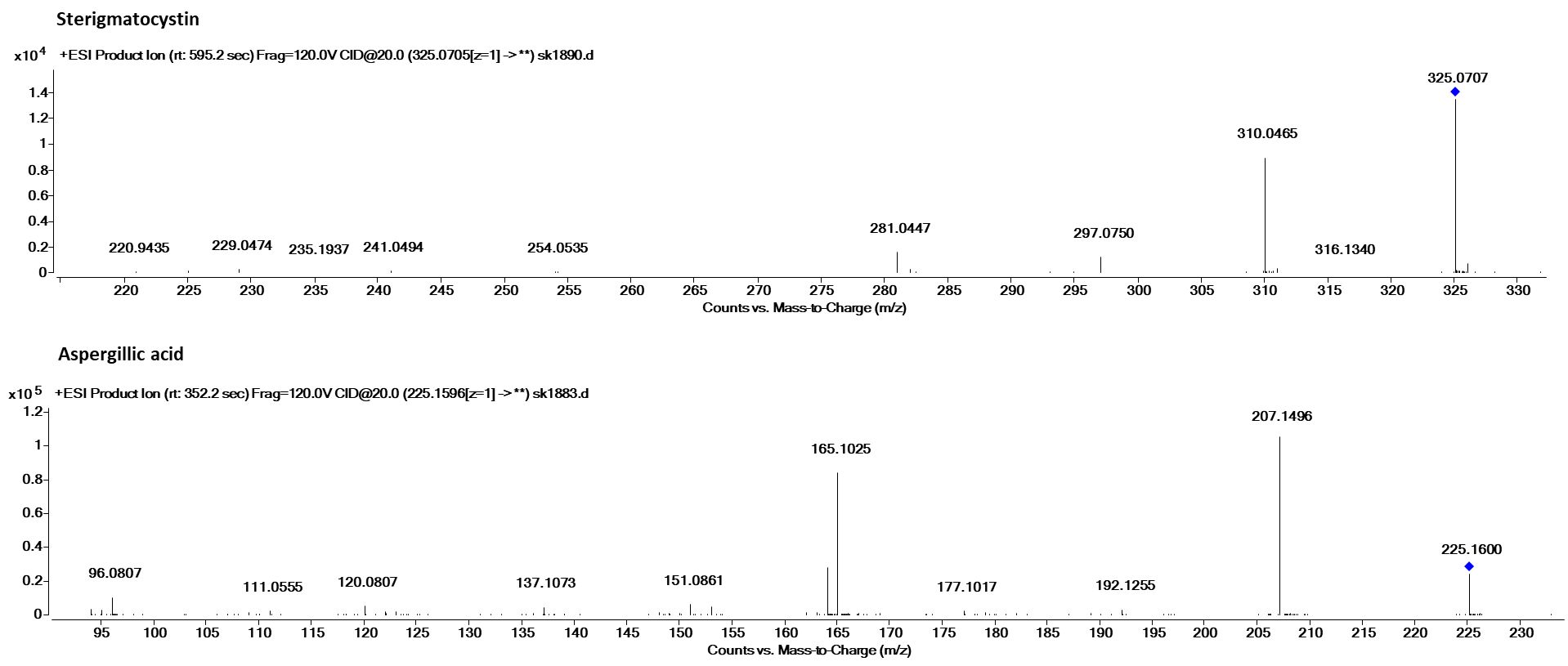


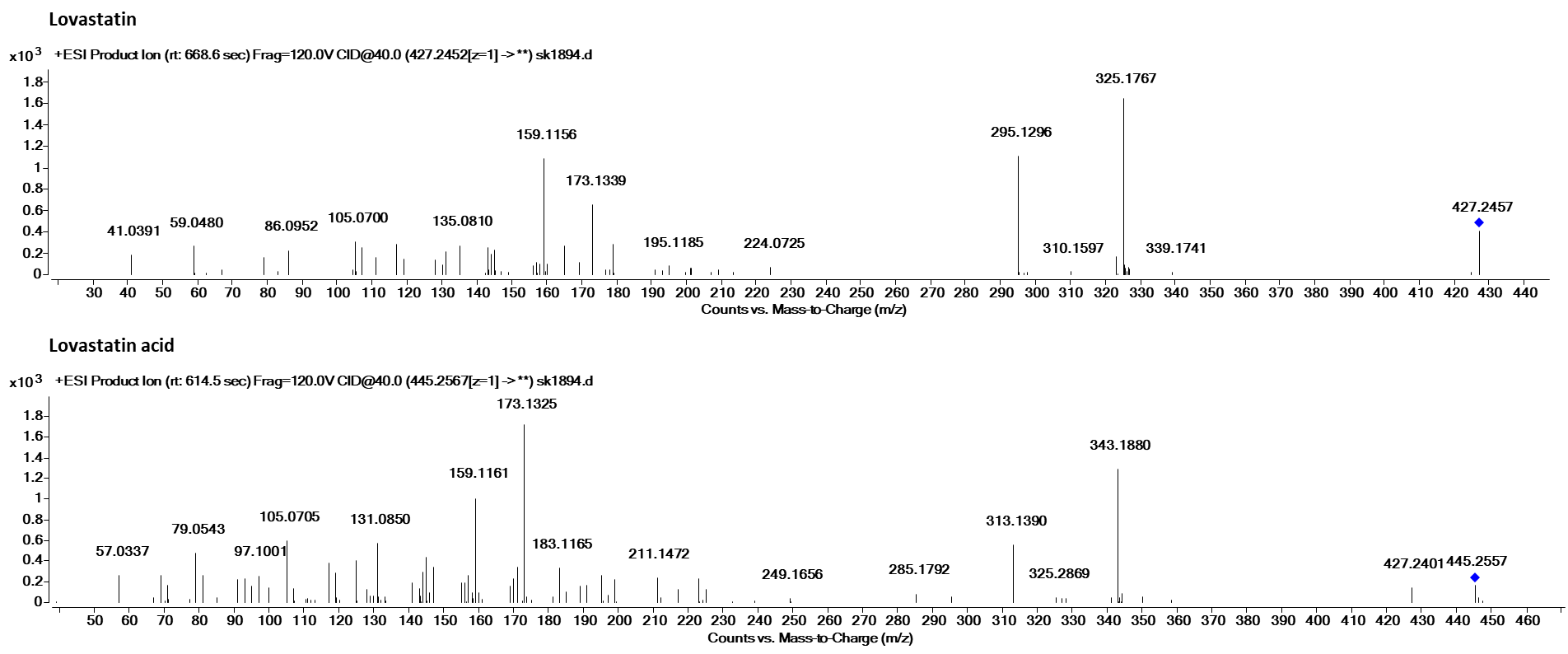


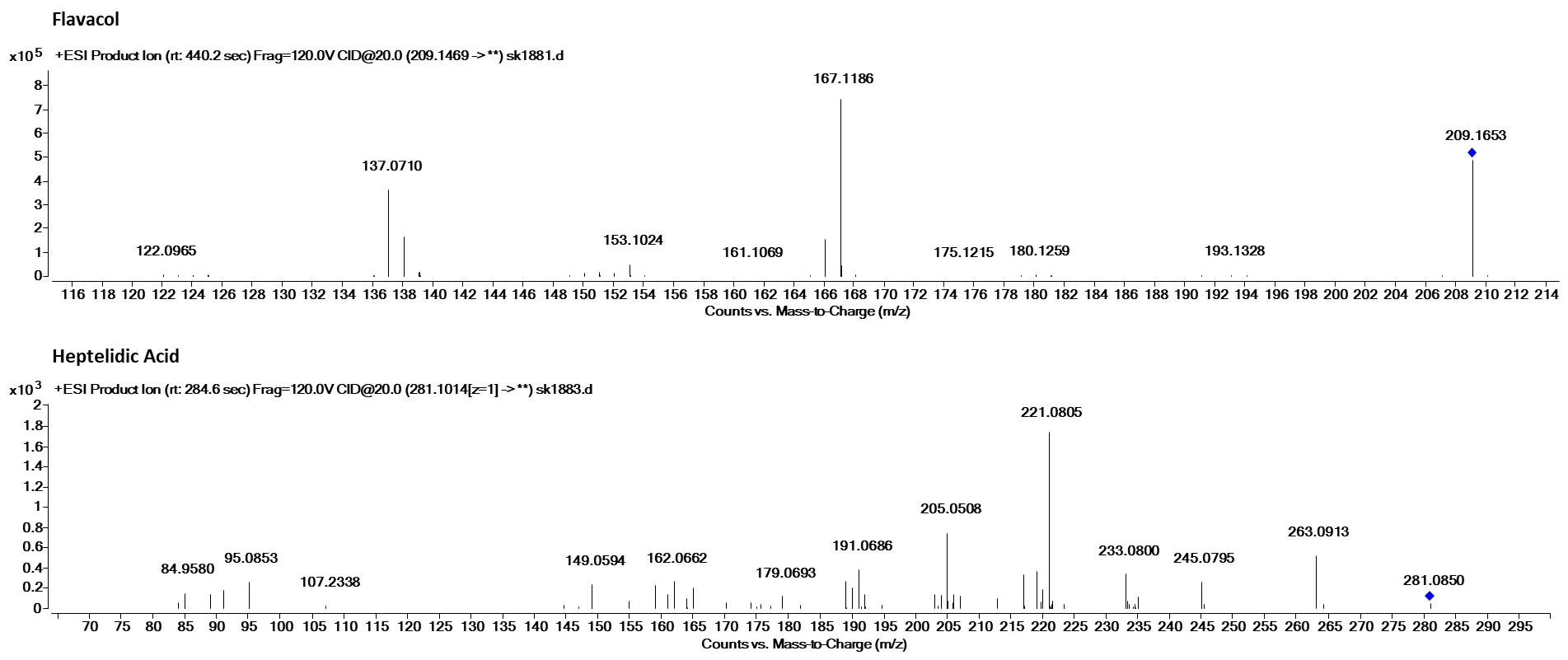


**Figure S11.** High resolution MS/MS spectra of some representative metabolites at 20 eV.
